# Impact of the COVID-19 Pandemic on the Personal Networks and Neurological Outcomes of People With Multiple Sclerosis: Cross-Sectional and Longitudinal Case-Control Study

**DOI:** 10.2196/45429

**Published:** 2024-02-06

**Authors:** Claire Riley, Shruthi Venkatesh, Amar Dhand, Nandini Doshi, Katelyn Kavak, Elle Levit, Christopher Perrone, Bianca Weinstock-Guttman, Erin Longbrake, Philip De Jager, Zongqi Xia

**Affiliations:** 1 Columbia University Irving Medical Center New York, NY United States; 2 University of Pittsburgh Pittsburgh, PA United States; 3 Brigham and Women's Hospital Boston, MA United States; 4 SUNY at Buffalo Buffalo, NY United States; 5 Yale University New Haven, CT United States; 6 University of Pennsylvania Philadelphia, PA United States

**Keywords:** neurology, neurodegenerative disease, multiple sclerosis, personal networks, COVID-19

## Abstract

**Background:**

The coronavirus disease 2019 (COVID-19) pandemic has negatively affected the social fabric.

**Objective:**

We evaluated the associations between personal social networks and neurological function in people with multiple sclerosis (pwMS) and controls in the prepandemic and pandemic periods.

**Methods:**

During the early pandemic (March-December 2020), 8 cohorts of pwMS and controls completed a questionnaire quantifying the structure and composition of their personal social networks, including the health behaviors of network members. Participants from 3 of the 8 cohorts had additionally completed the questionnaire before the pandemic (2017-2019). We assessed neurological function using 3 interrelated patient-reported outcomes: Patient Determined Disease Steps (PDDS), Multiple Sclerosis Rating Scale-Revised (MSRS-R), and Patient-Reported Outcomes Measurement Information System (PROMIS) Physical Function. We identified the network features associated with neurological function using paired 2-tailed *t* tests and covariate-adjusted regressions.

**Results:**

In the cross-sectional analysis of the pandemic data from 1130 pwMS and 1250 controls during the pandemic, having a higher percentage of network members with a perceived negative health influence was associated with worse disability in pwMS (MSRS-R: β=2.181, 95% CI 1.082-3.279; *P*<.001) and poor physical function in controls (PROMIS Physical Function: β=–5.707, 95% CI –7.405 to –4.010; *P*<.001). In the longitudinal analysis of 230 pwMS and 136 controls, the networks of all participants contracted, given an increase in constraint (pwMS-prepandemic: mean 52.24, SD 15.81; pwMS-pandemic: mean 56.77, SD 18.91; *P*=.006. Controls-prepandemic: mean 48.07, SD 13.36; controls-pandemic: mean 53.99, SD 16.31; *P*=.001) and a decrease in network size (pwMS-prepandemic: mean 8.02, SD 5.70; pwMS-pandemic: mean 6.63, SD 4.16; *P*=.003. Controls-prepandemic: mean 8.18, SD 4.05; controls-pandemic: mean 6.44, SD 3.92; *P*<.001), effective size (pwMS-prepandemic: mean 3.30, SD 1.59; pwMS-pandemic: mean 2.90, SD 1.50; *P*=.007. Controls-prepandemic: mean 3.85, SD 1.56; controls-pandemic: mean 3.40, SD 1.55; *P*=.01), and maximum degree (pwMS-prepandemic: mean 4.78, SD 1.86; pwMS-pandemic: mean 4.32, SD 1.92; *P*=.01. Controls-prepandemic: mean 5.38, SD 1.94; controls-pandemic: mean 4.55, SD 2.06; *P*<.001). These network changes were not associated with worsening function. The percentage of kin in the networks of pwMS increased (mean 46.06%, SD 29.34% to mean 54.36%, SD 30.16%; *P*=.003) during the pandemic, a change that was not seen in controls.

**Conclusions:**

Our findings suggest that high perceived negative health influence in the network was associated with worse function in all participants during the pandemic. The networks of all participants became tighter knit, and the percentage of kin in the networks of pwMS increased during the pandemic. Despite these perturbations in social connections, network changes from the prepandemic to the pandemic period were not associated with worsening function in all participants, suggesting possible resilience.

## Introduction

### Background

Multiple sclerosis (MS) is a chronic autoimmune disease affecting the central nervous system, leading to neurodegeneration and neurological disability [1,2]. Despite notable advancement in elucidating the factors influencing MS susceptibility, the mechanisms underlying disability accumulation are less well defined [3]. In addition to genetic predisposition, modifiable environmental factors such as personal networks play a key role in shaping health outcomes for people living with neurological diseases [4].

Personal social network features can affect the health outcomes of people living with neurological diseases, including stroke, traumatic brain injury, and MS [5-8]. In people with MS (pwMS), advantageous personal network structures (e.g., larger network size and more diffuse connections among network members) are associated with better language function and larger regional brain volume [9]. Conversely, adverse personal networks are associated with increased social isolation and loneliness, which may in turn exert direct biological effects by altering inflammation, recovery from injury, and resilience against neurodegeneration [10-18].

Our prior research implicates personal social networks as a potentially modifiable environmental contributor to neurological disability in pwMS [19,20]. Importantly, the vulnerability of pwMS to social isolation and loneliness exacerbated during the coronavirus disease 2019 (COVID-19) pandemic [21,22]. Physical distancing measures, stay-at-home orders, and travel restrictions imposed during the pandemic led to the contraction of personal social networks, increased social isolation and loneliness, excessive reliance on virtual communication, and alterations in the frequency and nature of social interactions [23-28]. Understanding how personal social network structure (e.g., network size and density) and composition (e.g., demographics and health behaviors of the network members) changed during the pandemic and assessing the impact of these changes on disability accumulation in MS could potentially inform novel interventions that may improve the quality of life and health outcomes for pwMS.

### Objectives

In this study, we compared the personal social networks of pwMS and controls during the COVID-19 pandemic and identified network features associated with increased disability accumulation. Further, we examined changes in personal social networks due to the COVID-19 pandemic in a subset of individuals with prepandemic data.

## Methods

### Study Cohort

During the pandemic, the multicenter Multiple Sclerosis Resilience to COVID-19 (MSReCOV) Collaborative recruited new pwMS and healthy controls through the University of Pittsburgh Medical Center (UPMC), Columbia University Irving Medical Center (CUIMC), University of Buffalo Medical Center, University of Pennsylvania, and Yale University [29-32]. In addition, pwMS and controls were recruited from 2 existing clinic-based cohorts (one at UPMC and another at CUIMC) and from a national cohort of first-degree relatives of pwMS (Genes and Environment in Multiple Sclerosis [GEMS] study) [33-38]. The inclusion criteria were adults aged ≥18 years either with or without a neurologist-confirmed diagnosis of MS.

For the cross-sectional analysis, we included pwMS and healthy controls from the MSReCOV study as well as 3 previously established cohorts (clinic cohorts at UPMC and CUIMC as well as the GEMS cohort). We deployed a modified personal network (PERSNET) questionnaire (Multimedia Appendix 1) between March 2020 and December 2020 to assess demographic, clinical, and personal social network features through a secure web-based platform (REDCap [Research Electronic Data Capture]) [39].

**Figure 1:**
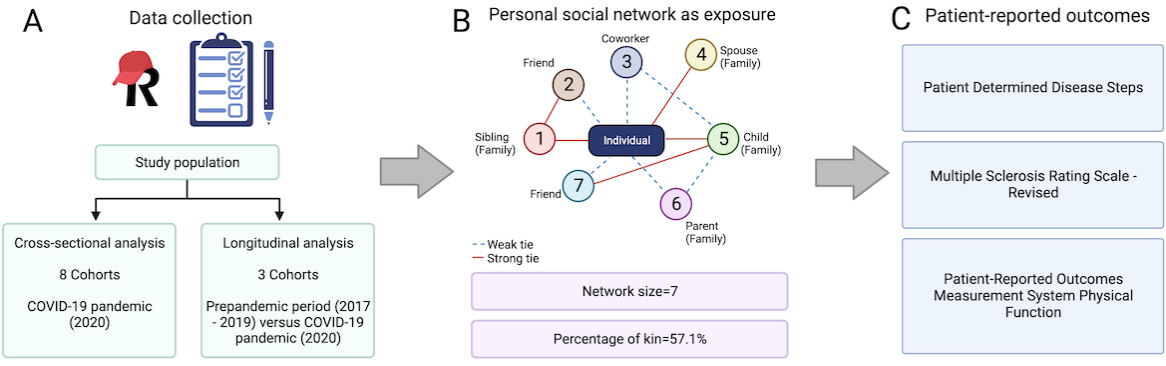
Overview of study design. (A) Schematic illustration of study populations and survey data collection using REDCap (Research Electronic Data Capture). (B) Personal social network features as exposure, as illustrated by a representative network of a hypothetical participant. (C) Patient-reported outcomes of neurological disability or physical function.

For the longitudinal analysis, we leveraged PERSNET data collected before the COVID-19 pandemic (2017-2019) from the 3 existing cohorts (clinic cohorts at UPMC and CUIMC as well as the GEMS cohort) for comparison with the early pandemic data in the same participants (Figure 1A) [20].

### Ethical Considerations

The institutional review board of each enrolling site approved the study (UPMC: STUDY21100060 and STUDY20040160; CUIMC: AAAS9668; University of Buffalo Medical Center: MOD00008107; University of Pennsylvania: 843454; and Yale University: HIC 2000027987). All participants provided informed consent. Participants completed the survey anonymously through a secure privacy-compliant platform [39]. Participation was voluntary. To ensure confidentiality, deidentified data were used for analysis. The study did not provide compensation for survey completion.

### Personal Network Metrics

We deployed an updated version of the PERSNET survey, adapted from the foundational General Social Survey [5-7,19,20,40]. In the PERSNET survey, participants identified individuals in their personal social network with whom they discuss personal matters or socialize or from whom they derive social support. These individuals could have any professional or personal relationship with the participant (e.g., coworker, parent, sibling, spouse, or child). We assessed the structure and composition of each participant’s network [41,42].

Network structure includes 6 quantitative features: size (number of individuals in the network, excluding the index person), density (sum of ties, excluding the index person’s ties, divided by all possible ties), constraint (a more granular density that assesses the extent to which the index person is connected to individuals who are connected to one another), effective size (number of nonredundant network members), and maximum degree and mean degree (highest and average number of ties, respectively, belonging to a network member).

Network composition quantifies the demographic characteristics and health behaviors of network members. Network demographics include the percentage of kin (percentage of network members who are family), SD of age (age range of network members), diversity of sex (proportion of sexes from 0 to 1, where 0 indicates a single sex and 1 indicates an equal ratio of men and women), and diversity of race (similar proportion of represented races, where 0 indicates a single race). Network health behaviors include the percentage of network members who smoke, consume alcohol, exhibit poor dietary habits, lead sedentary lifestyles, and exert perceived negative health influence. Other network composition features include the frequency, duration, and the living distance of network member from the index person, which quantify the depth of the relationships. Compositional features account for network size.

### Neurological Outcomes

To assess the status of neurological function, we used 3 interrelated patient-reported outcomes (PROs). The Patient Determined Disease Steps (PDDS) scale indicates the extent of gait impairment and correlates with the clinician-determined Expanded Disability Status Scale (EDSS). PDDS scores range from 0 to 8, where 0 corresponds to normal, and 8 indicates bedbound status [43]. The Multiple Sclerosis Rating Scale-Revised (MSRS-R) assesses the global neurological symptom burden, including walking, function in the extremities, vision, speech, swallowing, cognition, sensation, bladder function, and bowel function [44,45]. Each symptom domain score ranges from 0 to 4, where 0 indicates no impairment, and 4 indicates severe impairment. Higher cumulative MSRS-R scores (0-32) indicate greater neurological symptom burden and worse neurological function. Patient-Reported Outcomes Measurement Information System (PROMIS) Physical Function (version 1.2) is a generalizable measure of physical function also validated for pwMS [46,47]. PROMIS Physical Function is reported as a normally distributed T-score on a scale ranging from 0 to 100, where 50 represents the average for the US population and higher scores indicate better function. Whereas PDDS and MSRS-R are specific for pwMS, PROMIS is applicable to both pwMS and controls. All participants completed PRO assessment when completing the PERSNET survey during the pandemic, whereas a subset also completed the PROs before the pandemic.

### Covariates

We considered the following confounding factors that could potentially influence neurological function: age, sex, race, ethnicity, disease duration, employment, education, occupation, income, marital status, and cohabitant status. Race was categorized as African or African American, American Indian or Alaska Native or Native Hawaiian or other Pacific Islander, Asian, White, multiracial, or other. Ethnicity was categorized as Hispanic (or Latinx) and non-Hispanic. Because of the relatively small number of racial and ethnic minority participants in our cohorts, race and ethnicity were dichotomized as non-Hispanic White versus otherwise (encompassing individuals of Hispanic and non-European descent) in subsequent analyses. Disease duration was defined as the time from the first self-reported neurological symptom onset to the time of the most recent PRO assessment. Employment status was categorized as employed for wages, self-employed, out of work and looking for work, out of work but not currently looking for work, homemaker, student, military, retired, or unable to work. Education level was classified based on the highest level of education achieved: some high school or less, high school graduate, some college, associate degree, bachelor’s degree, or graduate degree. Occupation was categorized as business owner, executive or manager, professional, sales or clerical worker, service worker, or other. Annual household income level included the following brackets: ≤US $19,999, US $20,000 to US $34,999, US $35,000 to US $49,999, US $50,000 to US $64,999, US $65,000 to US $79,999, US $80,000 to US $94,999, US $95,000 to US $109,999, US $110,000 to US $124,999, and ≥US $125,000. Marital status was categorized as married or unmarried. Cohabitant status was categorized as living alone versus otherwise. In the subset with prepandemic data, the time elapsed between prepandemic and pandemic PERSNET assessments ranged from 1 to 4 years. Some covariates were missing because certain questions (e.g., employment status, income) in the PERSNET survey were made optional to reduce patient discomfort when completing the questionnaire.

To select the informative covariates, we examined the correlation between these features and PROs in univariate analyses (Figure S1 in Multimedia Appendix 2). We included features that met the following predefined criteria as covariates for downstream analyses: feature presence in >70% of pwMS, a Pearson correlation coefficient of ≥0.1, and a nominal statistical significance (*P*<.05) in association with all 3 PROs. We identified age, disease duration, employment, and income as meeting the criteria and adjusted these covariates in regression models involving pwMS. For analyses involving controls, we adjusted for age, employment, and income but not disease duration because it does not apply to controls. For the longitudinal analyses, we further adjusted for the time elapsed between prepandemic and pandemic PERSNET assessments as well as study cohort as additional covariates for consistency with our previous analysis [19,20].

### Statistical Analysis

We performed two types of analyses: (1) cross-sectional comparison between pwMS and controls during the COVID-19 pandemic and (2) longitudinal analysis of pwMS and controls during the COVID-19 pandemic when compared to their prepandemic baseline.

For the cross-sectional analysis, we first compared the personal networks of pwMS and controls using paired 2-tailed *t* tests. Next, we examined the association between network features (structure and composition) and PROs in pwMS in covariate-adjusted regression models. Given that the PROMIS Physical Function measure is generalizable across health and disease, we further assessed the association between network features and PROMIS Physical Function scores in controls for comparison. In a joint analysis of the pandemic data that include both pwMS and controls, we performed a moderation analysis to assess whether having an MS diagnosis influenced the association between network features and PROMIS Physical Function scores [48-50].

For the longitudinal analysis, we examined the *within-subject* differences in network features in pwMS and controls during the pandemic when compared to the most proximal prepandemic baseline (i.e., the closest value before the pandemic as baseline) using paired *t* tests. Next, we assessed the association of change in network features (i.e., pandemic value minus prepandemic baseline) in relation to the latest available PROs (during the pandemic) in pwMS using covariate-adjusted regressions and an omnibus test [20,38]. For the omnibus test, we combined the *P* values derived from the covariate-adjusted regressions for each PRO. Using the Fisher combined probability test, we calculated the chi-squared statistic and compared the observed values with the expected empirical distribution. To further interrogate these relationships, we generated a quantile-quantile (Q-Q) plot of the observed versus expected *P* values of the associations between the longitudinal changes in each network feature (pandemic value minus prepandemic baseline) and each PRO (the latest available score during the pandemic). The 95% CIs of the Q-Q plot were obtained from the empirical *P* value distribution generated by 10,000 permutations of the null hypothesis. We used 10,000 permutations, given the large number of network features and the sample size [19,20]. We performed similar longitudinal analysis in controls using PROMIS Physical Function for comparison. Raw *P* values were adjusted by Bonferroni correction for multiple comparisons. All statistical analyses were performed using R software (version 3.6.0) [51].

### Code Availability

The code for this project is available on GitHub [52].

## Results

### Participant Characteristics

The cross-sectional analysis included 1130 pwMS (age: mean 50.7, SD 12.1 y) and 1250 controls (age: mean 44.3, SD 12.1 y; Table 1). Participants were predominantly women (pwMS: 925/1130, 81.86%; controls: 960/1250, 76.80%) and non-Hispanic White (pwMS: 1043/1130, 92.30%; controls: 1208/1250, 96.64%). pwMS were less likely to be employed (544/1130, 48.14%) than controls (876/1250, 70.08%). The disability burden among pwMS was mild to moderate (PDDS score: mean 1.85, SD 2.12; MSRS-R score: mean 7.55, SD 5.49; PROMIS Physical Function score: mean 46.4, SD 10.82).

**Table 1:** Characteristics of the cross-sectional and longitudinal cohort participants.

Characteristics			Cross-sectional cohorts^a^				Longitudinal cohorts^b^				
			pwMS^c^ (N=1130)	Controls (N=1250)	*P* value^d^		pwMS (N=230)		Controls (N=136)	*P* value^d^	
Age (y), mean (SD)			50.7 (12.1)	44.4 (12.1)	*<.001*		50.4 (11.7)		43.1 (12.6)	*<.001*	
**Gender, n (%)**						*.009*					.17
	Woman		925 (81.9)	960 (76.8)			183 (79.6)		99 (72.8)		
	Man		203 (18.0)	286 (22.9)			47 (20.4)		37 (27.2)		
	Nonbinary intersex		2 (0.2)	4 (0.3)			0 (0)		0 (0)		
**Race, n (%)**						*<.001*					.18
	African or African American		46 (4.1)	12 (1)			7 (3)		0 (0)		
	American Indian or Alaska Native or Native Hawaiian or other Pacific Islander		10 (0.9)	6 (0.5)			1 (0.4)		0 (0)		
	Asian		8 (0.7)	8 (0.6)			1 (0.4)		1 (0.7)		
	White		1043 (92.7)	1208 (96.7)			214 (93.0)		135 (98.5)		
	Multiracial		9 (0.8)	11 (0.9)			4 (1.7)		1 (0.7)		
	Other		4 (0.4)	3 (0.2)			0 (0)		0 (0)		
	Not sure		5 (0.4)	1 (0.1)			3 (1.3)		0 (0)		
**Ethnicity, n (%)**						.26					.59
	Hispanic or Latinx		38 (3.4)	31 (2.5)			6 (2.6)		2 (1.5)		
	Non-Hispanic		1068 (94.9)	1201 (96.3)			220 (95.7)		131 (95.6)		
	Not sure		19 (1.7)	15 (1.2)			4 (1.7)		4 (2.9)		
**Education, n (%)**						*<.001*					*.004*
	High school graduate		70 (6.2)	35 (2.8)			12 (5.3)		2 (1.8)		
	Some college		154 (13.7)	106 (8.5)			30 (13.3)		4 (3.5)		
	Associate degree		111 (9.9)	70 (5.6)			20 (8.8)		5 (4.4)		
	Bachelor’s degree		363 (32.4)	441 (35.3)			75 (33.3)		53 (46.5)		
	Graduate degree		418 (37.3)	595 (47.6)			88 (39.1)		50 (43.9)		
**Employment^e^, n (%)**						*<.001*					*<.001*
	Employed for wages		544 (48.1)	876 (70.1)			98 (43.9)		81 (71.1)		
	Homemaker		63 (5.6)	77 (6.2)			11 (4.9)		5 (4.4)		
	Out of work and looking for work		17 (1.5)	27 (2.2)			8 (3.5)		5 (4.4)		
	Out of work but not currently looking for work		29 (2.6)	15 (1.2)			6 (2.7)		0 (0)		
	Retired		187 (16.5)	98 (7.8)			34 (15)		9 (7.9)		
	Self-employed		63 (5.6)	97 (7.8)			14 (6.2)		10 (8.8)		
	Student		12 (1.1)	18 (1.4)			3 (1.3)		2 (1.8)		
	Unable to work		190 (16.8)	39 (3.1)			49 (21.7)		2 (1.8)		
	Military		2 (0.2)	0 (0)			0 (0)		0 (0)		
**Annual household income (US $), n (%)**						*<.001*					*.004*
	≤19,999		74 (7.6)	37 (3.1)			19 (12.6)		5 (4.5)		
	20,000-34,999		85 (8.7)	56 (4.7)			15 (9.9)		2 (1.8)		
	35,000-49,999		97 (10)	75 (6.3)			23 (15.2)		9 (8.1)		
	50,000-64,999		92 (9.5)	106 (8.9)			14 (9.3)		11 (9.9)		
	65,000-79,999		79 (8.1)	120 (10.1)			7 (4.6)		8 (7.2)		
	80,000-94,999		76 (7.8)	94 (7.9)			8 (5.3)		8 (7.2)		
	95,000-109,999		80 (8.2)	127 (10.7)			12 (7.9)		14 (12.6)		
	110,000-124,999		85 (8.7)	112 (9.4)			20 (13.2)		12 (10.8)		
	≥125,000		303 (31.2)	461 (38.8)			33 (21.9)		42 (37.8)		
Married, n (%)			786 (69.9)	841 (67.4)	.22		146 (64)		96 (70.6)	.24	
Live alone, n (%)			156 (14.8)	186 (15)	.93		39 (17.1)		21 (15.4)	.79	
**Occupation, n (%)**						*.001*					.35
		Business owner	35 (6.2)	23 (2.4)			6 (8.1)		5 (5.5)		
		Executive or manager	100 (17.6)	157 (16.2)			14 (18.9)		8 (8.8)		
		Laborer or unskilled worker	7 (1.2)	4 (0.4)			0 (0)		0 (0)		
		Machine operator, inspector, or bus or cab driver	1 (0.2)	0 (0)			0 (0)		0 (0)		
		Mechanic, electrician, or skilled worker	6 (1.1)	14 (1.4)			0 (0)		0 (0)		
		Other	80 (14.1)	129 (13.3)			9 (12.2)		13 (14.3)		
		Professional	282 (49.6)	545 (56.1)			38 (51.4)		57 (62.6)		
		Sales or clerical worker	54 (9.5)	84 (8.6)			7 (9.5)		7 (7.7)		
		Service worker	3 (0.5)	16 (1.6)			0 (0)		1 (1.1)		
PDDS^f^ score, mean (SD)			1.9 (2.1)	N/A^g^	N/A		N/A		N/A	N/A	
MSRS-R^h^ score, mean (SD)			7.6 (5.5)	N/A	N/A		N/A		N/A	N/A	
PROMIS^i^ Physical Function T-score, mean (SD)			46.4 (10.8)	56.3 (9.0)	*<.001*		N/A		N/A	N/A	

^a^Cross-sectional cohorts include cohorts 1 and 2 (University of Pittsburgh Medical Center: clinic-based cohort and Multiple Sclerosis Resilience to COVID-19 [MSReCOV] collaborative recruitment), cohorts 3 and 4 (Columbia University Irving Medical Center: clinic-based cohort and MSReCOV collaborative recruitment), cohort 5 (Yale University), cohort 6 (University of Buffalo Medical Center), cohort 7 (University of Pennsylvania), and cohort 8 (Genes and Environment in Multiple Sclerosis [GEMS] cohort).

^b^Longitudinal cohorts include cohort 1 (University of Pittsburgh Medical Center: clinic-based cohort), cohort 3 (Columbia University Irving Medical Center: clinic-based cohort), and cohort 8 (GEMS cohort).

^c^pwMS: people with multiple sclerosis.

^d^Italicized *P* values met the significance threshold (*P*<.05).

^e^The sample size for certain questionnaire response may be smaller than the overall cohort size due to the optional completion of certain questions (e.g., employment status) to minimize participant discomfort.

^f^PDDS: Patient Determined Disease Steps.

^g^N/A: not applicable.

^h^MSRS-R: Multiple Sclerosis Rating Scale-Revised.

^i^PROMIS: Patient-Reported Outcomes Measurement Information System.

The longitudinal analysis included 230 pwMS and 136 controls. Similar to the cross-sectional analysis, controls were younger (age: mean 50.4, SD 11.7 y for pwMS and mean 43.1, SD 12.6 y for controls), had higher levels of education (163/225, 72.4% of pwMS and 103/114, 90.4% of controls completed college) and employment (pwMS: 98/223, 43.9%; controls: 81/114, 71.1%), and had higher annual household income than pwMS.

### Cross-Sectional Analysis of the Pandemic Period

First, we compared the network features of pwMS and controls during the COVID-19 pandemic (Table 2). In this unadjusted analysis, pwMS had higher density, higher constraint, smaller effective size, a higher percentage of kin, a lower percentage of people known for <6 years, lower percentage of network members who live >15 miles (>24 km) away, and lower percentage of network members who drink in their social networks when compared to controls. pwMS and controls reported similarly high percentages of their network members with a perceived negative health influence (pwMS: mean 36.16%, SD 30.43%; controls: mean 36.34%, SD 30.16%).

**Table 2:** Comparison of personal social network features in people with multiple sclerosis (MS) and controls during the COVID-19 pandemic.

Features		pwMS^a^ (N=1130), mean (SD)	Controls (N=1250), mean (SD)	*P* value ^b^
**Network structure**				
	Network size	6.79 (4.24)	6.78 (3.90)	.95
	Density	0.76 (0.25)	0.72 (0.24)	*<.001*
	Constraint	56.88 (19.07)	54.30 (17.03)	*.001*
	Effective size	2.97 (1.62)	3.38 (1.65)	*<.001*
	Maximum degree	4.38 (2.07)	4.54 (1.99)	.06
	Mean degree	3.48 (1.77)	3.43 (1.59)	.49
**Network composition**				
	Percentage of kin	55.89 (29.50)	50.71 (28.84)	*<.001*
	SD of age	12.65 (6.80)	11.96 (5.82)	.03
	Diversity of sex	67.26 (39.72)	70.80 (30.26)	.02
	Diversity of race	6.00 (16.42)	7.32 (17.03)	.06
	Percentage of network members contacted weekly or less	15.89 (20.90)	17.67 (21.15)	.04
	Percentage of network members known for <6 years	11.81 (19.61)	17.57 (23.65)	*<.001*
	Percentage of network members who live >15 miles (>24 km) away	32.45 (27.82)	38.93 (26.63)	*<.001*
	Percentage of network members who drink	12.43 (24.03)	16.63 (26.55)	*<.001*
	Percentage of network members who smoke	8.87 (18.14)	7.17 (15.85)	.02
	Percentage of network members who are nonexercisers	35.28 (31.60)	39.06 (30.43)	.004
	Percentage of network members who have a bad diet	23.04 (27.61)	22.75 (28.08)	.81
	Percentage of network members with a negative health influence	36.16 (30.43)	36.34 (30.16)	.89

^a^pwMS : people with multiple sclerosis.

^b^Italicized *P* values met the significance threshold (*P*<.002; α=.05, corrected for 18 comparisons).

Next, we examined the association between each structural and compositional network feature in relation to PDDS, MSRS-R, and PROMIS Physical Function scores in pwMS during the pandemic, after adjusting for age, disease duration, employment, and income in linear regression models (Table 3). pwMS who had a higher percentage of network members with a perceived negative health influence had higher MSRS-R scores, indicating greater MS symptom burden and disability (β=2.181, 95% CI 1.082-3.279; *P*<.001). None of the specific network health behavior features (e.g., smoking, alcohol use, bad diet, and sedentary lifestyle) that might be construed as a negative health influence showed a statistically significant association with MSRS-R scores. No other network feature had a significant association with any of the PROs after Bonferroni correction.

**Table 3:** Cross-sectional analysis of personal social network features in relation to patient-reported outcomes in people with multiple sclerosis during the COVID-19 pandemic.

Features		PDDS^a^				MSRS-R^b^				PROMIS^c^ Physical Function		
		Sample size, n^d^	β^e^ (95% CI)	*P* value^f^	Sample size, n^d^		β^e^ (95% CI)	*P* value	Sample size, n^d^		β^e^ (95% CI)	*P* value^f^
**Network structure**												
	Size	737	–0.017 (–0.050 to 0.016)	.31	737		–0.032 (–0.117 to 0.053)	.46	728		0.165 (0.001 to 0.330)	.049
	Density	704	–0.051 (–0.569 to 0.467)	.85	704		0.115 (–1.212 to 1.443)	.87	695		–0.369 (–2.933 to 2.194)	.78
	Constraint	704	–0.001 (–0.009 to 0.006)	.68	704		–0.004 (–0.023 to 0.014)	.63	695		–0.006 (–0.041 to 0.029)	.75
	Effective size	704	0.006 (–0.079 to 0.091)	.90	704		–0.044 (–0.262 to 0.173)	.69	695		0.150 (–0.270 to 0.569)	.48
	Maximum degree	704	0.018 (–0.050 to 0.085)	.60	704		0.020 (–0.153 to 0.192)	.82	695		0.017 (–0.316 to 0.350)	.92
	Mean degree	704	0.012 (–0.066 to 0.089)	.77	704		0.078 (–0.121 to 0.277)	.44	695		–0.049 (–0.433 to 0.335)	.80
**Network composition**												
	Percentage of kin	713	0.191 (–0.250 to 0.632)	.40	713		0.614 (–0.514 to 1.742)	.29	704		–1.356 (–3.541 to 0.830)	.22
	SD of age	534	–0.010 (–0.032 to 0.012)	.36	534		–0.013 (–0.071 to 0.045)	.66	526		0.025 (–0.082 to 0.132)	.65
	Diversity of sex	707	0.148 (–0.277 to 0.574)	.49	707		0.240 (–0.844 to 1.324)	.66	698		–1.143 (–3.249 to 0.962)	.29
	Diversity of race	701	0.545 (–0.403 to 1.492)	.26	701		–1.058 (–3.48 to 1.365)	.39	692		–1.506 (–6.195 to 3.183)	.53
	Percentage of network members contacted weekly or less	713	0.394 (–0.245 to 1.034)	.23	713		–0.601 (–2.238 to 1.036)	.47	704		–1.130 (–4.306 to 2.046)	.49
	Percentage of network members known for <6 years	713	–0.215 (–0.909 to 0.478)	.54	713		–0.050 (–1.825 to 1.725)	.96	704		1.326 (–2.11 to 4.763)	.45
	Percentage of network members who live >15 miles (>24 km) away	713	–0.199 (–0.667 to 0.269)	.40	713		0.176 (–1.023 to 1.374)	.77	704		2.306 (–0.008 to 4.619)	.05
	Percentage of network members who drink	713	–0.326 (–0.920 to 0.268)	.28	713		–0.391 (–1.912 to 1.130)	.61	704		2.979 (–0.003 to 5.960)	.05
	Percentage of network members who smoke	713	–0.139 (–0.882 to 0.603)	.71	713		1.046 (–0.853 to 2.944)	.28	704		–2.155 (–5.827 to 1.518)	.25
	Percentage of network members who are nonexercisers	713	–0.069 (–0.486 to 0.348)	.75	713		0.771 (–0.295 to 1.837)	.15	704		–0.281 (–2.351 to 1.788)	.79
	Percentage of network members who have a bad diet	713	0.048 (–0.437 to 0.534)	.85	713		1.242 (0.003 to 2.481)	.049	704		–2.794 (–5.189 to –0.400)	.02
	Percentage of network members with a negative health influence	713	–0.281 (–0.714 to 0.153)	.20	713		2.181 (1.082 to 3.279)	*<.001*	704		–0.650 (–2.795 to 1.494)	.55

^a^PDDS: Patient Determined Disease Steps.

^b^MSRS-R: Multiple Sclerosis Rating Scale-Revised.

^c^PROMIS: Patient-Reported Outcomes Measurement Information System.

^d^The smaller sample size in this analysis when compared to the overall cohort size was due to the requirement for participants who had complete data elements for network feature, neurological outcome, and *all* covariates.

^e^Adjusted for potential confounders (as identified in Figure S1 in Multimedia Appendix 2), including age, disease duration, employment, and income.

^f^Italicized *P* value met the significance threshold (*P*<.00092; α=.05, corrected for 54 comparisons).

For comparison, we examined the association between each network feature and PROMIS Physical Function scores during the pandemic in controls (Table S2 in Multimedia Appendix 2). We adjusted for age, employment, and income, whereas disease duration was not applicable for controls. We found that a lower maximum degree (β=0.383, 95% CI 0.141-0.626; *P*=.002), a higher percentage of network members who smoke (β=–4.846, 95% CI –7.919 to –1.774; *P*=.002), and a higher percentage of network members with a perceived negative health influence (β=–5.707, 95% CI –7.405 to –4.010; *P*<.001) were associated with lower PROMIS Physical Function scores (worse physical function) in controls. Given that the percentage of network members with a perceived negative health influence was associated with MSRS-R scores in pwMS and with PROMIS Physical Function scores in controls, respectively, this important compositional network feature that assesses the perception of negative health influences in the personal social network may contribute to physical impairment in both pwMS and controls.

### Moderation Analysis Assessing the Role of Having an MS Diagnosis

**Figure 2:**
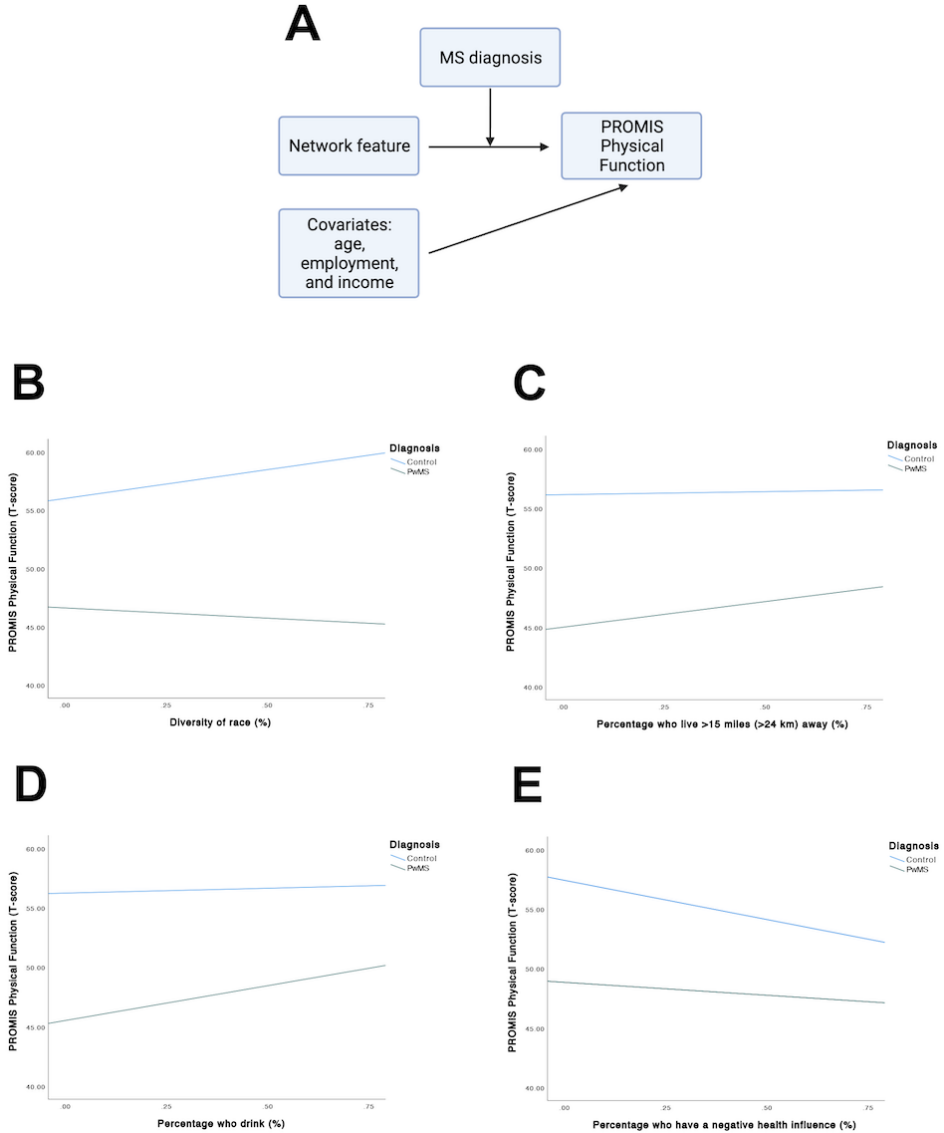
Role of multiple sclerosis (MS) diagnosis in moderating the association between personal social network features and Patient-Reported Outcomes Measurement Information System (PROMIS) Physical Function scores in people with MS (pwMS) and controls. (A) In a moderation analysis, we examined the association between each network feature and PROMIS Physical Function after combining the data from pwMS and controls and after adjusting for age, employment, and income as covariates and further investigated whether having an MS diagnosis moderated the direction or strength of this association. An MS diagnosis moderated the direction of the association between (B) diversity of race, (C) percentage who live >15 miles (>24 km) away, and (D) percentage who drink and PROMIS Physical Function, as well as the strength of the association between (E) percentage of people with a perceived negative health influence and PROMIS Physical Function. Values on x-axis indicate the 25th, 50th, 75th percentile.

We performed a moderation analysis to examine whether having an MS diagnosis influences the strength and direction of the association between network features and PROMIS Physical Function score during the COVID-19 pandemic (Figure 2A, Table S4 in Multimedia Appendix 2). In this joint analysis combining pwMS and controls, an MS diagnosis moderated the direction and strength of the association between several network features (diversity of race, percentage of network members who live >15 miles [>24 km] away, percentage of network members who drink, and percentage of network members with a perceived negative health influence) and PROMIS Physical Function score. For diversity of race, the moderating effect of an MS diagnosis was marginally significant (β=–5.878, 95% CI –10.889 to –0.868; *P*=.02) such that this network feature was not significantly associated with physical function in either pwMS (slope=–2.968, 95% CI –6.893 to 0.956; *P*=.14) or controls (slope=2.910, 95% CI –0.224 to 6.044; *P*=.07; Figure 2B). Having a higher percentage of network members who live >15 miles (>24 km) away was associated with higher physical function in pwMS (slope=3.890, 95% CI 1.852-5.928; *P*<.001) but not in controls (slope=–0.772, 95% CI –2.765 to 1.221; *P*=.45; Figure 2C), given the moderating effect of an MS diagnosis. Similarly, having a higher percentage of network members who drink was associated with worse physical function in pwMS (slope=3.716, 95% CI 1.392-6.039; *P*=.002) but again not in controls (slope=–0.982, 95% CI –2.966 to 1.003; *P*=.33; Figure 2D). For percentage of network members with a perceived negative health influence, both groups exhibited the same direction of association with physical function, but pwMS had a smaller magnitude of the association (slope=–2.174, 95% CI –4.019 to –0.329; *P*=.02) than controls (slope=–6.601, 95% CI –8.494 to –4.708; *P*<.001; Figure 2E).

### Longitudinal Analysis Comparing the Pandemic Period With the Prepandemic Baseline

We conducted a longitudinal analysis using a subset of pwMS and controls who completed the PERSNET survey both before and during the pandemic. During the pandemic, both pwMS and controls reported a lower percentage of people contacted weekly when compared to the prepandemic baseline (decrease from mean 23.48%, SD 24.95% to mean 14.89%, SD 24.95% for pwMS, *P*<.001; decrease from mean 30.34%, SD 26.35% to mean 18.78%, SD 22.2% for controls, *P*<.001), reflecting the widespread social isolation during the pandemic (Table 4). When compared to the prepandemic baseline in both pwMS and controls, there was a reduction in network size (pwMS-prepandemic: mean 8.02, SD 5.70; pwMS-pandemic: mean 6.63, SD 4.16; *P*=.003. Controls-prepandemic: mean 8.18, SD 4.05; controls-pandemic: mean 6.44, SD 3.92; *P*<.001), effective size (pwMS-prepandemic: mean 3.30, SD 1.59; pwMS-pandemic: mean 2.90, SD 1.50; *P*=.007. Controls-prepandemic: mean 3.85, SD 1.56; controls-pandemic: mean 3.40, SD 1.55; *P*=.01), and maximum degree (pwMS-prepandemic: mean 4.78, SD 1.86; pwMS-pandemic: mean 4.32, SD 1.92; *P*=.01. Controls-prepandemic: mean 5.38, SD 1.94; controls-pandemic: mean 4.55, SD 2.06; *P*<.001), as well as an increase in constraint (pwMS-prepandemic: mean 52.24, SD 15.81; pwMS-pandemic: mean 56.77, SD 18.91; *P*=.006. Controls-prepandemic: mean 48.07, SD 13.36; controls-pandemic: mean 53.99, SD 16.31; *P*=.001). These findings indicate contraction in personal social networks for both pwMS and controls during the pandemic period. There was an increase in the percentage of kin (from mean 46.06%, SD 29.34% to mean 54.36%, SD 30.16%; *P*=.003) in the networks of pwMS during the pandemic, which was not seen in controls.

**Figure 3:**
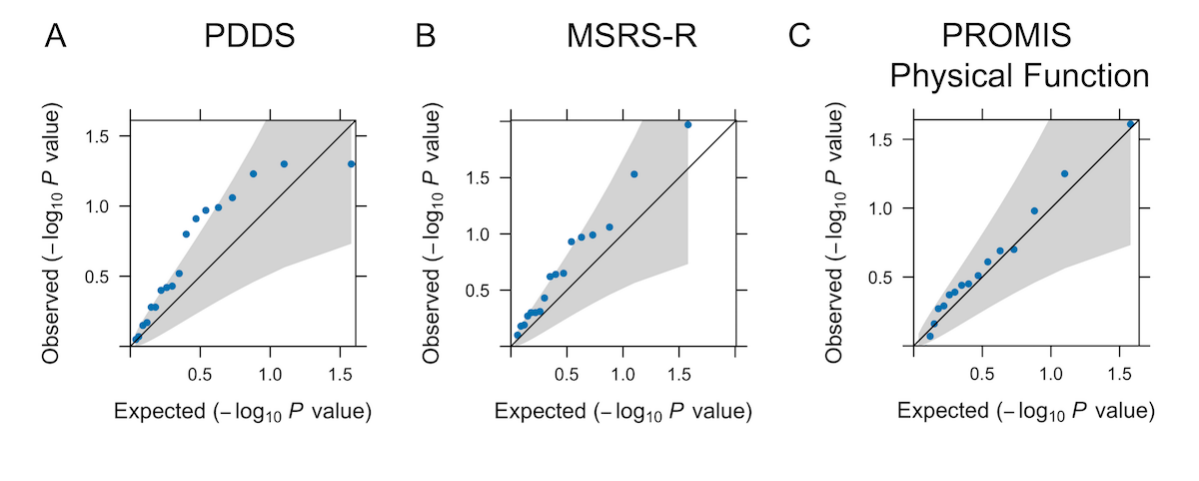
Quantile-quantile plots demonstrating longitudinal changes in personal social network features (due to the COVID-19 pandemic) in relation to patient-reported outcomes during the pandemic in people with multiple sclerosis. Comparison of observed and expected associations between the difference in each quantitative structural and compositional personal social network feature (pandemic value minus prepandemic baseline) in relation to each of the 3 measures of neurological and physical function status—(A) Patient Determined Disease Steps (PDDS), (B) Multiple Sclerosis Rating Scale-Revised (MSRS-R), and (C) Patient-Reported Outcomes Measurement Information System (PROMIS) Physical Function—during the pandemic in people with multiple sclerosis after adjusting for age, disease duration, employment, income, study cohort, and time lapse between prepandemic and pandemic personal network questionnaire (PERSNET) survey assessments. Expected *P* values (–log10 *P* value) were plotted on the x-axis and observed *P* values (–log10 *P* value) on the y-axis. The gray area encompasses the 95% CIs. Points outside the gray area were considered statistically significant without adjustment for multiple comparisons.

Finally, we examined whether changes in network features due to the pandemic (i.e., pandemic value minus the most proximal prepandemic baseline) in pwMS were associated with the PROs during the pandemic (Figure 3). We found no significant association between changes in network features and the latest available pandemic PROs. As a confirmation of these findings, there was no difference between the observed and expected distribution of the *P* values of association between changes in network features and pandemic PROs in the permuted omnibus test (PDDS: *P*=.88, MSRS-R: *P*=.29, and PROMIS Physical Function: *P*=.28).

**Table 4:** Personal social network features in people with multiple sclerosis (MS) and controls during the COVID-19 pandemic when compared to the within-subject prepandemic baseline.

Features		Controls: prepandemic baseline (N=136), mean (SD)	Controls: during the pandemic (N=136), mean (SD)	*P* value^a^	pwMS^b^: prepandemic baseline (N=230), mean (SD)	pwMS: during the pandemic (N=230), mean (SD)	*P* value
**Network structure**							
	Network size	8.18 (4.05)	6.44 (3.92)	*<.001*	8.02 (5.70)	6.63 (4.16)	*.003*
	Density	0.69 (0.24)	0.72 (0.24)	.24	0.74 (0.25)	0.77 (0.24)	.14
	Constraint	48.07 (13.36)	53.99 (16.31)	*.001*	52.24 (15.81)	56.77 (18.91)	*.006*
	Effective size	3.85 (1.56)	3.40 (1.55)	*.01*	3.30 (1.59)	2.90 (1.50)	*.007*
	Maximum degree	5.38 (1.94)	4.55 (2.06)	*<.001*	4.78 (1.86)	4.32 (1.92)	*.01*
	Mean degree	3.95 (1.71)	3.47 (1.72)	*.02*	3.70 (1.64)	3.47 (1.66)	.14
**Network composition**							
	Percentage of kin	47.69 (22.86)	50.27 (26.87)	.38	46.06 (29.34)	54.36 (30.16)	*.003*
	SD of age	12.67 (5.20)	11.60 (6.26)	.15	12.69 (6.05)	13.74 (6.69)	.23
	Diversity of sex	77.61 (24.00)	70.31 (30.73)	*.03*	57.14 (39.82)	52.17 (57.90)	.29
	Diversity of race	7.97 (18.55)	8.31 (18.43)	.87	5.66 (15.54)	7.13 (20.47)	.39
	Percentage of network members contacted weekly or less	30.34 (26.35)	18.78 (22.23)	*<.001*	23.48 (24.95)	14.89 (20.95)	*<.001*
	Percentage of network members known for <6 years	21.22 (23.58)	18.31 (21.95)	.27	13.26 (19.83)	10.41 (18.39)	.12
	Percentage of network members who live >15 miles (>24 km) away	41.37 (28.04)	38.88 (28.42)	.45	31.48 (25.57)	33.76 (29.36)	.38
	Percentage of network members who drink	17.85 (27.04)	19.13 (27.58)	.70	8.85 (18.87)	10.32 (21.66)	.51
	Percentage of network members who smoke	12.70 (24.28)	8.09 (18.57)	.06	12.72 (22.43)	9.13 (17.90)	.06
	Percentage of network members who are nonexercisers	41.93 (27.91)	39.86 (30.42)	.55	39.33 (30.94)	31.88 (33.13)	*.01*
	Percentage of network members who have a bad diet	31.32 (28.73)	25.74 (27.77)	.11	24.95 (29.57)	24.87 (30.21)	.98
	Percentage of network members who have a negative health influence	36.29 (24.75)	35.59 (25.14)	.82	36.77 (29.71)	38.02 (28.61)	.70

^a^Italicized *P* values met the significance threshold (*P*<.05).

^b^pwMS: people with multiple sclerosis.

## Discussion

### Principal Findings

Our quantitative assessment of the personal social network environment of pwMS during the early COVID-19 pandemic has several key findings. First, a higher percentage of network members with a perceived negative health influence was associated with greater neurological symptom burden in pwMS and worse physical function in controls during the pandemic, validating our prepandemic findings and suggesting a shared contribution of this important social environmental feature toward physical function in pwMS and controls. The magnitude of this association was larger in controls than in pwMS. Second, the personal social networks of pwMS and controls both experienced contraction during the COVID-19 pandemic when compared to the prepandemic baseline, and the personal networks of pwMS comprised a higher percentage of kin than those of controls during the pandemic. The percentage of people contacted weekly or less also decreased for both pwMS and controls, suggesting increased social isolation overall. Finally, changes in personal network features related to the COVID-19 pandemic (when compared to the prepandemic baseline) in pwMS were not associated with worsening disability during the pandemic, suggesting an element of neurological resilience despite the significant perturbation in social environment and connections.

Our study design has several novel aspects. First, this is the first known direct comparison of the personal social networks of pwMS and controls during the height of the COVID-19 pandemic when public health measures enacted to reduce contagion were widespread. Second, this is the first effort to longitudinally quantify changes in the personal networks of pwMS and controls due to the pandemic. Third, this study evaluated the differential impact of having an MS diagnosis on the associations between personal social network features and clinical outcomes in terms of both strength and directionality. Finally, this largest cross-sectional and longitudinal quantitative examination of the association of personal networks in relation to neurological and physical functions (not just in pwMS) explored the potential of neurological resilience secondary to social perturbation in the setting of the COVID-19 pandemic.

The finding of the association between a higher percentage of network members with a perceived negative health influence and worse neurological disability in pwMS and worse physical function in controls during the COVID-19 pandemic validated our prior findings from the prepandemic period [20]. These findings are unlikely to be spurious, given the relatively high proportion of participants (both pwMS and controls) whose network members have a perceived negative health influence. We hypothesize that having a higher percentage of network members with a perceived negative health influence in one’s social network could indicate low perceived social support and negative illness perception, which are both associated with worse psychosocial and health outcomes [53-59]. Moreover, this may decrease an individual’s likelihood of engaging in healthy behaviors (e.g., minimal alcohol consumption, abstention from smoking, regular exercise, healthy diet, and medication adherence) that may reduce overall comorbidities, MS-related disease activity, and neurological disability accumulation.

During the pandemic, the personal social networks of both pwMS and controls contracted when compared to the prepandemic period, as indicated by the decrease in network size, effective size, and maximum degree. Although changes in the density of the personal social networks were minimal, constraint increased in both pwMS and controls, providing further evidence for network contraction. The contraction of personal social networks was driven by the pruning of weak ties in the social network. In our analysis, the strength of the ties was quantified by assessing the frequency of interaction (percentage contacted weekly or less), the duration of contact (percentage known for <6 y), and the proximity (percentage who live >15 miles [>24 km] away) of network members. Although there was no change in the duration of contact or the proximity of network members for both pwMS and controls, there was a statistically significant decrease in the frequency of contact (percentage contacted weekly or less) in both groups. The reduction in the network size and frequency of contact suggests that both pwMS and control participants interacted less with weak ties in their network based on our method of assessment of tie strength. In the context of the early COVID-19 pandemic, it is conceivable that all participants kept their social circles tight knit and interacted predominantly with close contacts to minimize potential COVID-19 exposure.

The previously reported association between tightly knit personal social networks and worse physical function was not observed in this study [20]. We postulate several explanations. First, the contraction of social networks in pwMS as well as controls during the pandemic when compared to the prepandemic baseline is likely attributable to public health measures aiming to prevent the spread of COVID-19. Personal social networks contracted and became tighter knit among pwMS during the pandemic, whereas there was greater diversity (e.g., higher effective network size and lower constraint) in the personal networks of pwMS before the pandemic. In our prior cross-sectional study, the direction of the association between network features and physical function could not be fully determined [20]. It is possible that pwMS with better neurological and physical function might have had more diverse personal social networks before the pandemic, but the overall contraction of social networks during the pandemic among pwMS might have masked the association between tightly knit networks and worse physical function. Future longitudinal analyses examining the change in network features from the pandemic period to the postpandemic period may provide insight if the network diversity reverts to the prepandemic baseline versus if the new baseline persists. Second, because the contraction of personal social networks during the pandemic was a relatively recent (as of the data collection) and sudden event, there may not have been sufficient time for the onset of observable changes in neurological function. An assessment of neurological function at future time points could inform the long-term impact of social network changes due to the pandemic. Finally, the lack of an association between tight-knit personal networks and physical function suggests potential resilience of pwMS in coping with social changes attributable to the pandemic [57-60]. Although having tight-knit networks is typically associated with negative health outcomes, tight-knit networks may be advantageous in the context of minimizing COVID-19 exposures. In pwMS, these tight-knit networks saw an increase in the percentage of kin, which was not seen in controls. Having the support of kin could potentially help preserve neurological function in pwMS (e.g., family members help patients to get to clinic or rehabilitation visits or increase medication adherence). The differential impact of the COVID-19 pandemic and social isolation policies may have led pwMS to minimize social contact and restrict themselves to their tight-knit circle, given their higher risk of severe manifestations of COVID-19 [29-32].

The moderation analysis examined the interacting effect of having an MS diagnosis on the association between personal social networks and PROs in a joint analysis of pwMS and controls. An MS diagnosis moderated the association between specific personal social network features and physical function with respect to the direction (i.e., diversity of race, percentage of people who live >15 miles [>24 km] away, and percentage of people who drink) or strength (i.e., percentage of people with a perceived negative health influence) of the associations. Some of the differences in the results between the moderation analysis and the cross-sectional analysis for pwMS could be due to the inclusion of an MS diagnosis as a moderator variable (affecting the strength and direction of the association between network features and PROs) and inability to adjust for disease duration in this joint analysis because this covariate is not applicable for controls. Notably, a lower percentage of people who live >15 miles (>24 km) away and a lower percentage of people who drink within the personal networks of pwMS (but not controls) were each associated with worse physical function. pwMS with low physical function may need more support, which may explain their personal networks comprising a low percentage of people who live far away and a high proportion of kin who are more likely to live in the same household or in the vicinity. pwMS with low physical function may also be more inclined to seek out individuals whose healthy behaviors (e.g., minimal alcohol consumption) could have a positive health influence. The association between a higher percentage of people with a perceived negative health influence and worse physical function in pwMS (and controls) persisted. Interestingly, having higher perceived negative health influences had more of an impact on physical function in controls than in pwMS. We hypothesize that this effect is weaker in pwMS due to the likely resilience of pwMS, given their experience with a chronic neurological disease and having support from tight-knit circles, especially during the pandemic [60-63]. As pwMS have tight-knit networks with a high percentage of kin, it is conceivable that they have better support systems than controls. Interventions that empower individuals with the knowledge, skills, and support to effectively reduce the impact of perceived negative health influences (e.g., education on stress management and coping strategies) may be beneficial in the general population.

### Strengths and Limitations

This study has several strengths. First, the longitudinal analysis of changes in personal social networks due to the pandemic in comparison with the prepandemic baseline had a within-subject design. Consequently, we postulated that changes in network features suggestive of personal network contraction in the same participants during the pandemic (compared to their prepandemic baseline) is likely attributable to the pandemic, possibly because of the necessary public health measures. Second, we used three independent but interrelated PROs as a pragmatic method to assess the real-world status of neurological disability and physical function during the early pandemic period when clinical research participation became severely restricted. These validated PROs, two specifically for MS and one generalizable across health and disease, have shown strong correlations with physician-rated measures of neurological function (e.g., EDSS) in prior studies [43-47]. Third, we conducted both cross-sectional and longitudinal analyses in not only pwMS but also in controls, which enabled a comparison of the differential impact of the pandemic on their personal social networks. Fourth, the moderation analysis examining the role of having an MS diagnosis on the association between network features and physical function enabled an exploration of the strength and directionality of these complex relationships. Finally, we leveraged a large multicenter data set representative of the northeastern and mid-Atlantic regions of the United States with greater geographic diversity than prior studies, potentially increasing the generalizability of the study findings.

Our study also has limitations. First, the direction of the association between the percentage of people with a perceived negative health influence and physical function cannot be determined because we cannot infer causality. Therefore, it is possible that participants with greater disability or worse physical function could perceive that their personal network members exert a negative health influence. Conversely, participants with a higher percentage of network members with a perceived negative health influence could have greater disability or worse physical function. This limitation could be addressed by testing causality in future intervention studies. For example, in persons with a high percentage of people with a negative influence in the personal social network, we can compare the efficacy of interventions engaging the social network of the index person (e.g., providing closer monitoring of medication adherence and more encouragement to promote healthy behaviors) against the standard of care. Second, we did not assess the health status of network members as perceived by the index participant (e.g., if a network member was in bad or good health) or examine the association between the health status of the index participant and the perceived health status of their network members. This limitation could be addressed by incorporating relevant questions in future versions of the PERSNET survey. Third, the sample size of the longitudinal analysis was limited by the relatively modest number of participants with available quantified prepandemic personal networks as baseline. As such, the study did not have sufficient power for subgroup analysis stratified by demographic or clinical subtypes. Last, we sampled participants’ personal networks and neurological and functional status at one time point during the COVID-19 pandemic. The COVID-19 cases varied across areas and throughout the pandemic, whereas participants resided in urban, suburban, and rural environments that were differentially affected during the pandemic. Nevertheless, most of the study populations shared broader geographic regions (northeastern and mid-Atlantic United States) and completed the study response during the early period of the pandemic when federal- and state-level mandates were relatively uniform in terms of stay-at-home orders, physical distancing, and other mitigation guidelines.

### Conclusions

In conclusion, this study highlights the impact of the COVID-19 pandemic on personal social networks in pwMS and controls. Our findings generate important hypotheses for testing future interventions that may modify personal social networks to improve health outcomes. Future longitudinal studies examining the long-term impact of the COVID-19 pandemic on the evolution of personal social networks and neurological outcomes in people with chronic neurological disorders such as MS are warranted.

## Appendix

Multimedia Appendix 1 Personal network (PERSNET) survey.

Multimedia Appendix 2 Supplementary material.

## Abbreviations

COVID-19: coronavirus disease 2019
CUIMC: Columbia University Irving Medical Center
EDSS: Expanded Disability Status Scale
GEMS: Genes and Environment in Multiple Sclerosis
MS: multiple sclerosis
MSReCOV: Multiple Sclerosis Resilience to COVID-19
MSRS-R: Multiple Sclerosis Rating Scale-Revised
PDDS: Patient Determined Disease Steps
PERSNET: personal network
PRO: patient-reported outcome
PROMIS: Patient-Reported Outcomes Measurement Information System
pwMS: people with multiple sclerosis
REDCap: Research Electronic Data Capture
UPMC: University of Pittsburgh Medical Center

## Data Availability

The data sets generated for this study are not publicly available because protected health information is collected in the PERSNET survey. The deidentified data are available from the corresponding author on reasonable request and will be shared with approval from each participating site.
