# Supplementary material for: Impact of the COVID-19 Pandemic on the Personal Networks and Neurological Outcomes of People With Multiple Sclerosis: Cross-Sectional and Longitudinal Case-Control Study
Source: JMIR Public Health Surveill. 2024 Feb 6;10:e45429. doi: 10.2196/45429 (PMC10879979; doi:10.2196/45429)
Supplement: Multimedia Appendix 1 [file publichealth_v10i1e45429_app1.pdf]

Personal Network Survey

Please complete the survey below.

Thank you!

Welcome to the Personal Network Survey

This Personal Network Survey is a scientific approach to understand how your social ties influence achievement of your health-related goals.

We estimate that this survey will take about 20-25 minutes. The preferred platform for taking this survey is a desktop computer, laptop computer, or a tablet with external keyboard. You may also use a smartphone, though it may be more difficult for you to enter your answers.

Please carefully follow the instructions, particularly at the beginning of this survey, as your answers impact the remaining questions.

Please tell us a little bit about yourself.

Date of Birth

____
(Please do not click on the button for today's date unless it is applicable)

Your Age

____

Gender

◯ Male
◯ Female
◯ Other

Race (Select all that apply)

◯ African or African American
◯ American Indian or Alaskan Native
◯ Asian
◯ Caucasian
◯ Native Hawaiian or Other Pacific Islander
◯ Multi-racial
◯ Other
◯ Not Sure

Ethnicity

◯ Hispanic or Latino
◯ NOT Hispanic or Latino
◯ Not Sure

Education level

◯ Some high school or less
◯ High school grad
◯ Some college
◯ Associate degree
◯ Bachelor's degree
◯ Graduate degree
◯ Prefer not to answer

Current Address	__________________________________<br>(e.g."123 Main Street, New York, NY, 10032-1234")
Zip Code (9-digit):<br><br>Note: 9-digit zip code can be populated using the following link.<br>9-Digit LookUp	__________________________________<br>(e.g."10032-1234")
Employment Status	◯ Employed for wages<br>◯ Self-employed<br>◯ Out of work and looking for work<br>◯ Out of work but not currently looking for work<br>◯ A homemaker<br>◯ A student<br>◯ Military<br>◯ Retired<br>◯ Unable to work<br>◯ Prefer not to answer
Current Occupation	◯ Executive, manager<br>◯ Sales or clerical worker<br>◯ Mechanic, electrician, skilled worker<br>◯ Machine operator, inspector, bus/cab driver<br>◯ Service worker (e.g., janitor, guard)<br>◯ Professional (e.g., nurse, lawyer, teacher, etc.)<br>◯ Business owner<br>◯ Laborer, unskilled worker<br>◯ Farming<br>◯ Military<br>◯ Other
What was your total household income during the past 12 months?	◯ 0 to $19,999<br>◯ $20,000 to $34,999<br>◯ $35,000 to $49,999<br>◯ $50,000 to $64,999<br>◯ $65,000 to $79,999<br>◯ $80,000 to $94,999<br>◯ $95,000 to $109,999<br>◯ $110,000 to $124,999<br>◯ $125,000 or higher<br>(This includes your regular income.)
What is your approximate household net worth?	◯ less than $5,000<br>◯ $5,000 to $49,000<br>◯ $50,000 to $169,000<br>◯ $170,000 to $499,000<br>◯ more than $500,000<br>(This is the value of all the assets of people in your household (like housing, cars, stock, retirement funds, and business ownership) minus any debt or loans you and household members may have (like mortgage, credit card debt or car, school, or business loans). This does not include your regular income.)
Marital Status	◯ Not married<br>◯ Married

Do you live alone?

◯ Yes
◯ No

How many people are in your household?

____

Who is in your Personal Network?

In order to understand your personal network and the different ways in which people are important to you and support you, we are now going to help you create a master list of people by asking you who falls into these three categories:

Who do you discuss important personal matters with?Who do you often socialize with?Who provides you support for your health needs?
We are only interested in people who fall into one of those three categories, and we'd like you to think only about your current network of people in your life who are age 18 or older.

Please only use first names, nicknames, or initials - DO NOT USE FULL NAMES. If two people have the same first name, then add a last initial. You can enter any identifier you'd like in place of a name - all that matters is that you know the person you mean, as the "names" you enter will be used later in the survey.

Who do you discuss personal matters with?

Name 1

____

Name 2

____

Name 3

____

Name 4

____

Name 5

____

More names, separated by commas (if needed):

____

Who do you often socialize with? For example, these are people with whom you go out to dinner, go on a trip, or hang out.

Please do not list full names, and don't worry about duplicates from the previous section.

Name 1 ______________________

Name 2 ______________________

Name 3 ______________________

Name 4 ______________________

Name 5 ______________________

More names, separated by commas (if needed): ____________________________

Think about people who encourage you to stay healthy by giving you motivation, advice, or direct help. Who provides this kind of support for your health?

If applicable, please include your caregiver(s). A caregiver is a spouse or partner, family member or friend who provides emotional support, assistance, and care as needed (note that this person may receive payment in their role as a caregiver).

Please do not list full names, and don't worry about duplicates from the previous section.

Name 1 ______________________

Name 2 ______________________

Name 3 ______________________

Name 4 ______________________

Name 5 ______________________

More names, separated by commas (if needed): ____________________________

Finalizing your list of people:

Now, we are going to create a master list from the names you provided above, and you will remove duplicates and blanks. Know that we are only going to use the first 5 names you entered from each section above - that's okay!

Please read these instructions carefully:Click the "Keep" button next to the first time a person is named in the list. If you do not click "Keep," then the name will be removed.If a person shows up in the list more than once, only choose "Keep" once.All blanks and duplicates should be left as "Remove".

	Keep	Remove
[name1]	◯	◯
[name2]	◯	◯
[name3]	◯	◯
[name4]	◯	◯
[name5]	◯	◯
[name6]	◯	◯
[name7]	◯	◯
[name8]	◯	◯
[name9]	◯	◯
[name10]	◯	◯
[name11]	◯	◯
[name12]	◯	◯
[name13]	◯	◯
[name14]	◯	◯
[name15]	◯	◯

Number Filled in First 10 Slots

____
(This is just for us to keep track.)

Total number of social contacts

____
(Total number of names you kept.)

Relationship Description

We are now going to focus on the relationships in your social network. If you notice some names missing, that is okay - we are only using the first 10 from your master list.

Below should be a list of unique names without blanks. If there are missing names from the first 10, duplicate names, or blanks, please STOP and go back to the previous section where you made your master list by choosing "Keep" for certain names. Please review the instructions for that section, and adjust your "Keep" or "Remove" choices as needed.

We are interested in how close you feel to each person below. Compared to everyone you've listed, how close do you feel to each person?

[name1]	◯ especially close<br>◯ not especially close
[name2]	◯ especially close<br>◯ not especially close
[name3]	◯ especially close<br>◯ not especially close
[name4]	◯ especially close<br>◯ not especially close
[name5]	◯ especially close<br>◯ not especially close
[name6]	◯ especially close<br>◯ not especially close
[name7]	◯ especially close<br>◯ not especially close
[name8]	◯ especially close<br>◯ not especially close
[name9]	◯ especially close<br>◯ not especially close
[name10]	◯ especially close<br>◯ not especially close
[name11]	◯ especially close<br>◯ not especially close
[name12]	◯ especially close<br>◯ not especially close
[name13]	◯ especially close<br>◯ not especially close
[name14]	◯ especially close<br>◯ not especially close
[name15]	◯ especially close<br>◯ not especially close

We will now focus on the relationship between each pair of people you mentioned. For example, we will ask about [name1] and [name2]. For each pair, we will ask whether they are total strangers, in-between, or especially close. Here is what we mean by each term:

Total strangers: The two people wouldn't recognize one another if they met on the street.

In-between: Relationships in the middle of total strangers and especially close. Typically, these people are casual acquaintances.

Especially close: The two people are as close or closer to each other than they are to you.

Take your time. This can be difficult.

Is [name1] a total stranger, especially close, or in-between with [name2]?	◯ stranger<br>◯ in-between<br>◯ especially close
Is [name1] a total stranger, especially close, or in-between with [name3]?	◯ stranger<br>◯ in-between<br>◯ especially close
Is [name1] a total stranger, especially close, or in-between with [name4]?	◯ stranger<br>◯ in-between<br>◯ especially close
Is [name1] a total stranger, especially close, or in-between with [name5]?	◯ stranger<br>◯ in-between<br>◯ especially close
Is [name1] a total stranger, especially close, or in-between with [name6]?	◯ stranger<br>◯ in-between<br>◯ especially close
Is [name1] a total stranger, especially close, or in-between with [name7]?	◯ stranger<br>◯ in-between<br>◯ especially close
Is [name1] a total stranger, especially close, or in-between with [name8]?	◯ stranger<br>◯ in-between<br>◯ especially close
Is [name1] a total stranger, especially close, or in-between with [name9]?	◯ stranger<br>◯ in-between<br>◯ especially close
Is [name1] a total stranger, especially close, or in-between with [name10]?	◯ stranger<br>◯ in-between<br>◯ especially close
Is [name1] a total stranger, especially close, or in-between with [name11]?	◯ stranger<br>◯ in-between<br>◯ especially close

Is [name1] a total stranger, especially close, or in-between with [name12]?	◯ stranger<br>◯ in-between<br>◯ especially close
Is [name1] a total stranger, especially close, or in-between with [name13]?	◯ stranger<br>◯ in-between<br>◯ especially close
Is [name1] a total stranger, especially close, or in-between with [name14]?	◯ stranger<br>◯ in-between<br>◯ especially close
Is [name1] a total stranger, especially close, or in-between with [name15]?	◯ stranger<br>◯ in-between<br>◯ especially close

Is [name2] a total stranger, especially close, or in-between with [name3]?	◯ stranger<br>◯ in-between<br>◯ especially close
Is [name2] a total stranger, especially close, or in-between with [name4]?	◯ stranger<br>◯ in-between<br>◯ especially close
Is [name2] a total stranger, especially close, or in-between with [name5]?	◯ stranger<br>◯ in-between<br>◯ especially close
Is [name2] a total stranger, especially close, or in-between with [name6]?	◯ stranger<br>◯ in-between<br>◯ especially close
Is [name2] a total stranger, especially close, or in-between with [name7]?	◯ stranger<br>◯ in-between<br>◯ especially close
Is [name2] a total stranger, especially close, or in-between with [name8]?	◯ stranger<br>◯ in-between<br>◯ especially close
Is [name2] a total stranger, especially close, or in-between with [name9]?	◯ stranger<br>◯ in-between<br>◯ especially close
Is [name2] a total stranger, especially close, or in-between with [name10]?	◯ stranger<br>◯ in-between<br>◯ especially close
Is [name2] a total stranger, especially close, or in-between with [name11]?	◯ stranger<br>◯ in-between<br>◯ especially close
Is [name2] a total stranger, especially close, or in-between with [name12]?	◯ stranger<br>◯ in-between<br>◯ especially close

Is [name2] a total stranger, especially close, or in-between with [name13]?	◯ stranger<br>◯ in-between<br>◯ especially close
Is [name2] a total stranger, especially close, or in-between with [name14]?	◯ stranger<br>◯ in-between<br>◯ especially close
Is [name2] a total stranger, especially close, or in-between with [name15]?	◯ stranger<br>◯ in-between<br>◯ especially close

Is [name3] a total stranger, especially close, or in-between with [name4]?	◯ stranger<br>◯ in-between<br>◯ especially close
Is [name3] a total stranger, especially close, or in-between with [name5]?	◯ stranger<br>◯ in-between<br>◯ especially close
Is [name3] a total stranger, especially close, or in-between with [name6]?	◯ stranger<br>◯ in-between<br>◯ especially close
Is [name3] a total stranger, especially close, or in-between with [name7]?	◯ stranger<br>◯ in-between<br>◯ especially close
Is [name3] a total stranger, especially close, or in-between with [name8]?	◯ stranger<br>◯ in-between<br>◯ especially close
Is [name3] a total stranger, especially close, or in-between with [name9]?	◯ stranger<br>◯ in-between<br>◯ especially close
Is [name3] a total stranger, especially close, or in-between with [name10]?	◯ stranger<br>◯ in-between<br>◯ especially close
Is [name3] a total stranger, especially close, or in-between with [name11]?	◯ stranger<br>◯ in-between<br>◯ especially close
Is [name3] a total stranger, especially close, or in-between with [name12]?	◯ stranger<br>◯ in-between<br>◯ especially close
Is [name3] a total stranger, especially close, or in-between with [name13]?	◯ stranger<br>◯ in-between<br>◯ especially close
Is [name3] a total stranger, especially close, or in-between with [name14]?	◯ stranger<br>◯ in-between<br>◯ especially close

Is [name3] a total stranger, especially close, or in-between with [name15]?	◯ stranger<br>◯ in-between<br>◯ especially close

Is [name4] a total stranger, especially close, or in-between with [name5]?	◯ stranger<br>◯ in-between<br>◯ especially close
Is [name4] a total stranger, especially close, or in-between with [name6]?	◯ stranger<br>◯ in-between<br>◯ especially close
Is [name4] a total stranger, especially close, or in-between with [name7]?	◯ stranger<br>◯ in-between<br>◯ especially close
Is [name4] a total stranger, especially close, or in-between with [name8]?	◯ stranger<br>◯ in-between<br>◯ especially close
Is [name4] a total stranger, especially close, or in-between with [name9]?	◯ stranger<br>◯ in-between<br>◯ especially close
Is [name4] a total stranger, especially close, or in-between with [name10]?	◯ stranger<br>◯ in-between<br>◯ especially close
Is [name4] a total stranger, especially close, or in-between with [name11]?	◯ stranger<br>◯ in-between<br>◯ especially close
Is [name4] a total stranger, especially close, or in-between with [name12]?	◯ stranger<br>◯ in-between<br>◯ especially close
Is [name4] a total stranger, especially close, or in-between with [name13]?	◯ stranger<br>◯ in-between<br>◯ especially close
Is [name4] a total stranger, especially close, or in-between with [name14]?	◯ stranger<br>◯ in-between<br>◯ especially close
Is [name4] a total stranger, especially close, or in-between with [name15]?	◯ stranger<br>◯ in-between<br>◯ especially close

Is [name5] a total stranger, especially close, or in-between with [name6]?	◯ stranger<br>◯ in-between<br>◯ especially close

Is [name5] a total stranger, especially close, or in-between with [name7]?	◯ stranger<br>◯ in-between<br>◯ especially close
Is [name5] a total stranger, especially close, or in-between with [name8]?	◯ stranger<br>◯ in-between<br>◯ especially close
Is [name5] a total stranger, especially close, or in-between with [name9]?	◯ stranger<br>◯ in-between<br>◯ especially close
Is [name5] a total stranger, especially close, or in-between with [name10]?	◯ stranger<br>◯ in-between<br>◯ especially close
Is [name5] a total stranger, especially close, or in-between with [name11]?	◯ stranger<br>◯ in-between<br>◯ especially close
Is [name5] a total stranger, especially close, or in-between with [name12]?	◯ stranger<br>◯ in-between<br>◯ especially close
Is [name5] a total stranger, especially close, or in-between with [name13]?	◯ stranger<br>◯ in-between<br>◯ especially close
Is [name5] a total stranger, especially close, or in-between with [name14]?	◯ stranger<br>◯ in-between<br>◯ especially close
Is [name5] a total stranger, especially close, or in-between with [name15]?	◯ stranger<br>◯ in-between<br>◯ especially close

Is [name6] a total stranger, especially close, or in-between with [name7]?	◯ stranger<br>◯ in-between<br>◯ especially close
Is [name6] a total stranger, especially close, or in-between with [name8]?	◯ stranger<br>◯ in-between<br>◯ especially close
Is [name6] a total stranger, especially close, or in-between with [name9]?	◯ stranger<br>◯ in-between<br>◯ especially close
Is [name6] a total stranger, especially close, or in-between with [name10]?	◯ stranger<br>◯ in-between<br>◯ especially close
Is [name6] a total stranger, especially close, or in-between with [name11]?	◯ stranger<br>◯ in-between<br>◯ especially close

Is [name6] a total stranger, especially close, or in-between with [name12]?	◯ stranger<br>◯ in-between<br>◯ especially close
Is [name6] a total stranger, especially close, or in-between with [name13]?	◯ stranger<br>◯ in-between<br>◯ especially close
Is [name6] a total stranger, especially close, or in-between with [name14]?	◯ stranger<br>◯ in-between<br>◯ especially close
Is [name6] a total stranger, especially close, or in-between with [name15]?	◯ stranger<br>◯ in-between<br>◯ especially close

Is [name7] a total stranger, especially close, or in-between with [name8]?	◯ stranger<br>◯ in-between<br>◯ especially close
Is [name7] a total stranger, especially close, or in-between with [name9]?	◯ stranger<br>◯ in-between<br>◯ especially close
Is [name7] a total stranger, especially close, or in-between with [name10]?	◯ stranger<br>◯ in-between<br>◯ especially close
Is [name7] a total stranger, especially close, or in-between with [name11]?	◯ stranger<br>◯ in-between<br>◯ especially close
Is [name7] a total stranger, especially close, or in-between with [name12]?	◯ stranger<br>◯ in-between<br>◯ especially close
Is [name7] a total stranger, especially close, or in-between with [name13]?	◯ stranger<br>◯ in-between<br>◯ especially close
Is [name7] a total stranger, especially close, or in-between with [name14]?	◯ stranger<br>◯ in-between<br>◯ especially close
Is [name7] a total stranger, especially close, or in-between with [name15]?	◯ stranger<br>◯ in-between<br>◯ especially close

Is [name8] a total stranger, especially close, or in-between with [name9]?	◯ stranger<br>◯ in-between<br>◯ especially close

Is [name8] a total stranger, especially close, or in-between with [name10]?	◯ stranger<br>◯ in-between<br>◯ especially close
Is [name8] a total stranger, especially close, or in-between with [name11]?	◯ stranger<br>◯ in-between<br>◯ especially close
Is [name8] a total stranger, especially close, or in-between with [name12]?	◯ stranger<br>◯ in-between<br>◯ especially close
Is [name8] a total stranger, especially close, or in-between with [name13]?	◯ stranger<br>◯ in-between<br>◯ especially close
Is [name8] a total stranger, especially close, or in-between with [name14]?	◯ stranger<br>◯ in-between<br>◯ especially close
Is [name8] a total stranger, especially close, or in-between with [name15]?	◯ stranger<br>◯ in-between<br>◯ especially close

Is [name9] a total stranger, especially close, or in-between with [name10]?	◯ stranger<br>◯ in-between<br>◯ especially close
Is [name9] a total stranger, especially close, or in-between with [name11]?	◯ stranger<br>◯ in-between<br>◯ especially close
Is [name9] a total stranger, especially close, or in-between with [name12]?	◯ stranger<br>◯ in-between<br>◯ especially close
Is [name9] a total stranger, especially close, or in-between with [name13]?	◯ stranger<br>◯ in-between<br>◯ especially close
Is [name9] a total stranger, especially close, or in-between with [name14]?	◯ stranger<br>◯ in-between<br>◯ especially close
Is [name9] a total stranger, especially close, or in-between with [name15]?	◯ stranger<br>◯ in-between<br>◯ especially close

Is [name10] a total stranger, especially close, or in-between with [name11]?	◯ stranger<br>◯ in-between<br>◯ especially close

Is [name10] a total stranger, especially close, or in-between with [name12]?	◯ stranger<br>◯ in-between<br>◯ especially close
Is [name10] a total stranger, especially close, or in-between with [name13]?	◯ stranger<br>◯ in-between<br>◯ especially close
Is [name10] a total stranger, especially close, or in-between with [name14]?	◯ stranger<br>◯ in-between<br>◯ especially close
Is [name10] a total stranger, especially close, or in-between with [name15]?	◯ stranger<br>◯ in-between<br>◯ especially close
Is [name11] a total stranger, especially close, or in-between with [name12]?	◯ stranger<br>◯ in-between<br>◯ especially close
Is [name11] a total stranger, especially close, or in-between with [name13]?	◯ stranger<br>◯ in-between<br>◯ especially close
Is [name11] a total stranger, especially close, or in-between with [name14]?	◯ stranger<br>◯ in-between<br>◯ especially close
Is [name11] a total stranger, especially close, or in-between with [name15]?	◯ stranger<br>◯ in-between<br>◯ especially close
Is [name12] a total stranger, especially close, or in-between with [name13]?	◯ stranger<br>◯ in-between<br>◯ especially close
Is [name12] a total stranger, especially close, or in-between with [name14]?	◯ stranger<br>◯ in-between<br>◯ especially close
Is [name12] a total stranger, especially close, or in-between with [name15]?	◯ stranger<br>◯ in-between<br>◯ especially close
Is [name13] a total stranger, especially close, or in-between with [name14]?	◯ stranger<br>◯ in-between<br>◯ especially close

Is [name13] a total stranger, especially close, or in-between with [name15]?	◯ stranger<br>◯ in-between<br>◯ especially close
Is [name14] a total stranger, especially close, or in-between with [name15]?	◯ stranger<br>◯ in-between<br>◯ especially close

About People in Your Network

We are going to ask you a series of questions about each person in your network. In some cases, you may not know the exact answer for sure. Whenever possible, we would appreciate if you would guess - try to use the "don't know" category as little as possible.

Which person or persons support you most often? You can choose more than one person.

	Supports me most often
[name1]	◯
[name2]	◯
[name3]	◯
[name4]	◯
[name5]	◯
[name6]	◯
[name7]	◯
[name8]	◯
[name9]	◯
[name10]	◯
[name11]	◯
[name12]	◯
[name13]	◯
[name14]	◯
[name15]	◯

What kind of support does each person give you? (Select all that apply. You can choose more than one option)

Types of Support

Emotional Support: This would be someone who you turn to when you need help managing personal situations, and share feelings of happiness or sadness.Life Advice: This would be someone who gives you advice on life decisions such as employment or places to live.Financial Support: This would be someone who gives you advice or provides direct help with your daily or overall finances.Physical or Health Support: This would be someone who gives you advice on or provides direct help with medications/supplements, choosing doctors or getting to appointments, mental health resources, managing health insurance, or physical assistance around your household.Camaraderie: This would be someone who provides support through understanding important experiences that you have had together.

	Emotional support	Life advice	Financial support	Physical or Health support	Camaraderie	None
[name1]	☐	☐	☐	☐	☐	☐
[name2]	☐	☐	☐	☐	☐	☐
[name3]	☐	☐	☐	☐	☐	☐
[name4]	☐	☐	☐	☐	☐	☐
[name5]	☐	☐	☐	☐	☐	☐
[name6]	☐	☐	☐	☐	☐	☐
[name7]	☐	☐	☐	☐	☐	☐
[name8]	☐	☐	☐	☐	☐	☐
[name9]	☐	☐	☐	☐	☐	☐
[name10]	☐	☐	☐	☐	☐	☐
[name11]	☐	☐	☐	☐	☐	☐
[name12]	☐	☐	☐	☐	☐	☐
[name13]	☐	☐	☐	☐	☐	☐
[name14]	☐	☐	☐	☐	☐	☐
[name15]	☐	☐	☐	☐	☐	☐

	Male	Female	Other
[name1]	◯	◯	◯
[name2]	◯	◯	◯
[name3]	◯	◯	◯
[name4]	◯	◯	◯
[name5]	◯	◯	◯
[name6]	◯	◯	◯
[name7]	◯	◯	◯

[name8]	◯	◯	◯
[name9]	◯	◯	◯
[name10]	◯	◯	◯
[name11]	◯	◯	◯
[name12]	◯	◯	◯
[name13]	◯	◯	◯
[name14]	◯	◯	◯
[name15]	◯	◯	◯

Do any people in your network have a negative influence on your health? For example, some people can passively or actively encourage you to smoke, not eat well, or not exercise.

	Yes	No
[name1]	◯	◯
[name2]	◯	◯
[name3]	◯	◯
[name4]	◯	◯
[name5]	◯	◯
[name6]	◯	◯
[name7]	◯	◯
[name8]	◯	◯
[name9]	◯	◯
[name10]	◯	◯
[name11]	◯	◯
[name12]	◯	◯
[name13]	◯	◯
[name14]	◯	◯
[name15]	◯	◯

What is the race of each person in your social network?

	Black or African American	White	American Indian/Alaska Native	Asian	Native Hawaiian or Other Pacific Islander	Other	Don't know
[name1]	◯	◯	◯	◯	◯	◯	◯
[name2]	◯	◯	◯	◯	◯	◯	◯
[name3]	◯	◯	◯	◯	◯	◯	◯
[name4]	◯	◯	◯	◯	◯	◯	◯
[name5]	◯	◯	◯	◯	◯	◯	◯
[name6]	◯	◯	◯	◯	◯	◯	◯
[name7]	◯	◯	◯	◯	◯	◯	◯

[name8]	○	○	○	○	○	○	○
[name9]	○	○	○	○	○	○	○
[name10]	○	○	○	○	○	○	○
[name11]	○	○	○	○	○	○	○
[name12]	○	○	○	○	○	○	○
[name13]	○	○	○	○	○	○	○
[name14]	○	○	○	○	○	○	○
[name15]	○	○	○	○	○	○	○

What is the ethnicity of each person in your social network?

	Not Hispanic or Latino	Hispanic or Latino	Don't know
[name1]	○	○	○
[name2]	○	○	○
[name3]	○	○	○
[name4]	○	○	○
[name5]	○	○	○
[name6]	○	○	○
[name7]	○	○	○
[name8]	○	○	○
[name9]	○	○	○
[name10]	○	○	○
[name11]	○	○	○
[name12]	○	○	○
[name13]	○	○	○
[name14]	○	○	○
[name15]	○	○	○

As far as you know, what is the highest level of education of each person?

	Some high school or less	High school grad	Some college	Associate degree	Bachelor's degree	Graduate degree	Don't know
[name1]	○	○	○	○	○	○	○
[name2]	○	○	○	○	○	○	○
[name3]	○	○	○	○	○	○	○
[name4]	○	○	○	○	○	○	○
[name5]	○	○	○	○	○	○	○
[name6]	○	○	○	○	○	○	○
[name7]	○	○	○	○	○	○	○
[name8]	○	○	○	○	○	○	○
[name9]	○	○	○	○	○	○	○
[name10]	○	○	○	○	○	○	○

[name11]	○	○	○	○	○	○	○
[name12]	○	○	○	○	○	○	○
[name13]	○	○	○	○	○	○	○
[name14]	○	○	○	○	○	○	○
[name15]	○	○	○	○	○	○	○

On average, how often do you communicate with each person in your network?

	Daily	Weekly	Monthly	Less often	Don't know
[name1]	○	○	○	○	○
[name2]	○	○	○	○	○
[name3]	○	○	○	○	○
[name4]	○	○	○	○	○
[name5]	○	○	○	○	○
[name6]	○	○	○	○	○
[name7]	○	○	○	○	○
[name8]	○	○	○	○	○
[name9]	○	○	○	○	○
[name10]	○	○	○	○	○
[name11]	○	○	○	○	○
[name12]	○	○	○	○	○
[name13]	○	○	○	○	○
[name14]	○	○	○	○	○
[name15]	○	○	○	○	○

For how many years have you known the following people?

	Less than three	Three to six	More than six	Don't know
[name1]	○	○	○	○
[name2]	○	○	○	○
[name3]	○	○	○	○
[name4]	○	○	○	○
[name5]	○	○	○	○
[name6]	○	○	○	○
[name7]	○	○	○	○
[name8]	○	○	○	○
[name9]	○	○	○	○
[name10]	○	○	○	○
[name11]	○	○	○	○
[name12]	○	○	○	○
[name13]	○	○	○	○
[name14]	○	○	○	○
[name15]	○	○	○	○

We are now going to ask all the ways each person is connected to you. Some people can be connected to you in more than one way. For example, a man could be your brother, and he could belong to your church, and be your lawyer.

Please also indicate which individuals in your social network you consider as a caregiver. A caregiver is a spouse or partner, family member or friend who provides emotional support, assistance, and care as needed (note that this person may receive payment in their role as a caregiver).

For each person below, in what way are they connected to you? (Select all that apply)

	Spouse	Family	Friend	Advisor	Co - worker	Caregiver	Other
[name1]	☐	☐	☐	☐	☐	☐	☐
[name2]	☐	☐	☐	☐	☐	☐	☐
[name3]	☐	☐	☐	☐	☐	☐	☐
[name4]	☐	☐	☐	☐	☐	☐	☐
[name5]	☐	☐	☐	☐	☐	☐	☐
[name6]	☐	☐	☐	☐	☐	☐	☐
[name7]	☐	☐	☐	☐	☐	☐	☐
[name8]	☐	☐	☐	☐	☐	☐	☐
[name9]	☐	☐	☐	☐	☐	☐	☐
[name10]	☐	☐	☐	☐	☐	☐	☐
[name11]	☐	☐	☐	☐	☐	☐	☐
[name12]	☐	☐	☐	☐	☐	☐	☐
[name13]	☐	☐	☐	☐	☐	☐	☐
[name14]	☐	☐	☐	☐	☐	☐	☐
[name15]	☐	☐	☐	☐	☐	☐	☐

How old are the people in your social network?

[name1] ______________________

[name2] ______________________

[name3] ______________________

[name4] ______________________

[name5] ______________________

[name6] ______________________

[name7]	______________________
[name8]	______________________
[name9]	______________________
[name10]	______________________
[name11]	______________________
[name12]	______________________
[name13]	______________________
[name14]	______________________
[name15]	______________________

Health Habits

Please remember that the more carefully you can answer the following questions about yourself and those in your network, the more you are helping this research.

Have you cut back on heavy drinking of alcohol in the past 3 months?	◯ Yes<br>◯ No<br>◯ I do not drink heavily
Have you cut back on smoking in the past 3 months?	◯ Yes<br>◯ No<br>◯ I do not smoke
Have you exercised at least 3-4 times a week over the past 3 months?	◯ Yes<br>◯ No
Did you eat a healthy diet regularly over the past 3 months?	◯ Yes<br>◯ No
In which areas do you feel you experience more health problems than the average person? (Select all that apply)	☐ General Health<br>☐ Pain<br>☐ Cognitive/Mental Health<br>☐ Cardiac<br>☐ No Health Problems

Which people in your network do you think have cut back on heavy drinking of alcohol in the past 3 months?

	Yes	No	Does not drink heavily	Don't know
[name1]	◯	◯	◯	◯
[name2]	◯	◯	◯	◯
[name3]	◯	◯	◯	◯
[name4]	◯	◯	◯	◯
[name5]	◯	◯	◯	◯
[name6]	◯	◯	◯	◯
[name7]	◯	◯	◯	◯
[name8]	◯	◯	◯	◯
[name9]	◯	◯	◯	◯
[name10]	◯	◯	◯	◯
[name11]	◯	◯	◯	◯
[name12]	◯	◯	◯	◯
[name13]	◯	◯	◯	◯
[name14]	◯	◯	◯	◯
[name15]	◯	◯	◯	◯

Which people in your network do you think have cut back on smoking in the past 3 months?

	Yes	No	Does not smoke	Don't know
[name1]	◯	◯	◯	◯
[name2]	◯	◯	◯	◯
[name3]	◯	◯	◯	◯
[name4]	◯	◯	◯	◯
[name5]	◯	◯	◯	◯
[name6]	◯	◯	◯	◯
[name7]	◯	◯	◯	◯
[name8]	◯	◯	◯	◯
[name9]	◯	◯	◯	◯
[name10]	◯	◯	◯	◯
[name11]	◯	◯	◯	◯
[name12]	◯	◯	◯	◯
[name13]	◯	◯	◯	◯
[name14]	◯	◯	◯	◯
[name15]	◯	◯	◯	◯

Which people in your network do you think have exercised at least 3-4 times a week in the past 3 months?

	Yes	No	Don't know
[name1]	○	○	○
[name2]	○	○	○
[name3]	○	○	○
[name4]	○	○	○
[name5]	○	○	○
[name6]	○	○	○
[name7]	○	○	○
[name8]	○	○	○
[name9]	○	○	○
[name10]	○	○	○
[name11]	○	○	○
[name12]	○	○	○
[name13]	○	○	○
[name14]	○	○	○
[name15]	○	○	○

Which people in your network do you think ate a healthy diet regularly over the past 3 months?

	Yes	No	Don't know
[name1]	○	○	○
[name2]	○	○	○
[name3]	○	○	○
[name4]	○	○	○
[name5]	○	○	○
[name6]	○	○	○
[name7]	○	○	○
[name8]	○	○	○
[name9]	○	○	○
[name10]	○	○	○
[name11]	○	○	○
[name12]	○	○	○
[name13]	○	○	○
[name14]	○	○	○
[name15]	○	○	○

In which areas do people in your network experience health problems that are more than usual for an average person? (Select all that apply)

	General Health	Pain	Cognitive/Mental Health	Cardiac	No Health Problems	Don't know
[name1]	☐	☐	☐	☐	☐	☐
[name2]	☐	☐	☐	☐	☐	☐
[name3]	☐	☐	☐	☐	☐	☐
[name4]	☐	☐	☐	☐	☐	☐
[name5]	☐	☐	☐	☐	☐	☐
[name6]	☐	☐	☐	☐	☐	☐
[name7]	☐	☐	☐	☐	☐	☐
[name8]	☐	☐	☐	☐	☐	☐
[name9]	☐	☐	☐	☐	☐	☐
[name10]	☐	☐	☐	☐	☐	☐
[name11]	☐	☐	☐	☐	☐	☐
[name12]	☐	☐	☐	☐	☐	☐
[name13]	☐	☐	☐	☐	☐	☐
[name14]	☐	☐	☐	☐	☐	☐
[name15]	☐	☐	☐	☐	☐	☐

How far does each person live from you? For people less than 1 mile, click on 1-5 miles.

	Same house	1-5 miles	6-15 miles	16-50 miles	50+ miles
[name1]	○	○	○	○	○
[name2]	○	○	○	○	○
[name3]	○	○	○	○	○
[name4]	○	○	○	○	○
[name5]	○	○	○	○	○
[name6]	○	○	○	○	○
[name7]	○	○	○	○	○
[name8]	○	○	○	○	○
[name9]	○	○	○	○	○
[name10]	○	○	○	○	○
[name11]	○	○	○	○	○
[name12]	○	○	○	○	○
[name13]	○	○	○	○	○
[name14]	○	○	○	○	○
[name15]	○	○	○	○	○

Are any of the following people in your social network your first-degree relative(s) (i.e., full biological parent, sibling, or child)?

	Yes	No
[name1]	◯	◯
[name2]	◯	◯
[name3]	◯	◯
[name4]	◯	◯
[name5]	◯	◯
[name6]	◯	◯
[name7]	◯	◯
[name8]	◯	◯
[name9]	◯	◯
[name10]	◯	◯
[name11]	◯	◯
[name12]	◯	◯
[name13]	◯	◯
[name14]	◯	◯
[name15]	◯	◯

Do any of the following people in your social network have Multiple Sclerosis (MS)?

	Yes	No	Not Sure
[name1]	◯	◯	◯
[name2]	◯	◯	◯
[name3]	◯	◯	◯
[name4]	◯	◯	◯
[name5]	◯	◯	◯
[name6]	◯	◯	◯
[name7]	◯	◯	◯
[name8]	◯	◯	◯
[name9]	◯	◯	◯
[name10]	◯	◯	◯
[name11]	◯	◯	◯
[name12]	◯	◯	◯
[name13]	◯	◯	◯
[name14]	◯	◯	◯
[name15]	◯	◯	◯

Are you a caregiver for any of the following people?

	Yes	No	Not Sure
[name1]	◯	◯	◯
[name2]	◯	◯	◯
[name3]	◯	◯	◯
[name4]	◯	◯	◯
[name5]	◯	◯	◯
[name6]	◯	◯	◯
[name7]	◯	◯	◯
[name8]	◯	◯	◯
[name9]	◯	◯	◯
[name10]	◯	◯	◯
[name11]	◯	◯	◯
[name12]	◯	◯	◯
[name13]	◯	◯	◯
[name14]	◯	◯	◯
[name15]	◯	◯	◯

Have any of the following people in your social network tested positive for COVID19?

	Yes	No	Not Sure
[name1]	◯	◯	◯
[name2]	◯	◯	◯
[name3]	◯	◯	◯
[name4]	◯	◯	◯
[name5]	◯	◯	◯
[name6]	◯	◯	◯
[name7]	◯	◯	◯
[name8]	◯	◯	◯
[name9]	◯	◯	◯
[name10]	◯	◯	◯
[name11]	◯	◯	◯
[name12]	◯	◯	◯
[name13]	◯	◯	◯
[name14]	◯	◯	◯
[name15]	◯	◯	◯

Your Diagnosis

Have YOU been diagnosed with Multiple Sclerosis(MS)?

◯ Yes
◯ No

What is the date of your MS diagnosis?

____
(Please do not click on the button for today's date unless it is applicable)

At what age were you diagnosed with MS?

____

What is the date of your MS symptom onset?

____
(Please do not click on the button for today's date unless it is applicable)

Some people with MS do not feel comfortable sharing the fact that they have MS with other people within and outside of their social network. For each of the people in your social network, please indicate whether he/she knows or does not know that you have MS.

	Knows	Does not know
[name1]	◯	◯
[name2]	◯	◯
[name3]	◯	◯
[name4]	◯	◯
[name5]	◯	◯
[name6]	◯	◯
[name7]	◯	◯
[name8]	◯	◯
[name9]	◯	◯
[name10]	◯	◯
[name11]	◯	◯
[name12]	◯	◯
[name13]	◯	◯
[name14]	◯	◯
[name15]	◯	◯

For some people, keeping their MS diagnosis from people inside and/or outside of their social network can be stressful. Below is a list of people that you have indicated are unaware of your MS diagnosis. For each person, pelase indicate the degree to which not sharing your MS diagnosis is stressful for you.

	Not Stressful	Somewhat Stressful	Very Stressful
[name1]	◯	◯	◯
[name2]	◯	◯	◯

[name3]	◯	◯	◯
[name4]	◯	◯	◯
[name5]	◯	◯	◯
[name6]	◯	◯	◯
[name7]	◯	◯	◯
[name8]	◯	◯	◯
[name9]	◯	◯	◯
[name10]	◯	◯	◯
[name11]	◯	◯	◯
[name12]	◯	◯	◯
[name13]	◯	◯	◯
[name14]	◯	◯	◯
[name15]	◯	◯	◯

Your Functional Status

Below is a scale that attempts to rate YOUR abilities and disabilities. Please rate YOUR CURRENT functioning for each category.

	0 No symptoms: "I have no symptoms or disability in this specific area"	1 Some symptoms, no disability: "I am aware of symptoms but no limits on my activities"	2 Mild disability: "I have mild limits on my activities, but I do not need help from others or to use other aides"	3 Moderate disability: "I have moderate limits on my activities and I sometimes need help from others or use other aides"	4 Severe disability: "I have severe limits on my activities and I usually need help from others or use other aides"
Walking	◯	◯	◯	◯	◯
Using your arms and hands	◯	◯	◯	◯	◯
Vision	◯	◯	◯	◯	◯
Speech	◯	◯	◯	◯	◯
Swallowing	◯	◯	◯	◯	◯
Thinking, memory or cognition	◯	◯	◯	◯	◯
Numbness, tingling, burning sensation or pain	◯	◯	◯	◯	◯
Controlling your bladder and/or bowel	◯	◯	◯	◯	◯

Your Mobility

Patient Determined Disease Steps V2

The following section focuses mainly on how well you walk, regardless of your diagnosis.

Please read the choices listed and choose the one that best describes your own situation. If you feel that your situation is very different from any of the choices below, choose the "Unclassifiable".

Please select option 'Normal' if you have no mobility issues.

- ◯ 0 Normal: I may have some mild symptoms, mostly sensory due to MS but they do not limit my activity. If I do have an attack, I return to normal when the attack has passed.
- ◯ 1 Mild Disability: I have some noticeable symptoms from my MS but they are minor and have only a small effect on my lifestyle.
- ◯ 2 Moderate Disability: I don't have any limitations in my walking ability. However, I do have significant problems due to MS that limit daily activities in other ways.
- ◯ 3 Gait Disability: MS does interfere with my activities, especially my walking. I can work a full day, but athletic or physically demanding activities are more difficult than they used to be. I usually don't need a cane or other assistance to walk, but I might need some assistance during an attack.
- ◯ 4 Early Cane: I use a cane or a single crutch or some other form of support (such as touching a wall or leaning on someone's arm) for walking all the time or part of the time, especially when walking outside. I think I can walk 25 feet in 20 seconds without a cane or crutch. I always need some assistance (cane or crutch) if I want to walk as far as 3 blocks.
- ◯ 5 Late Cane: To be able to walk 25 feet, I have to have a cane, crutch or someone to hold onto. I can get around the house or other buildings by holding onto furniture or touching the walls for support. I may use a scooter or wheelchair if I want to go greater distances.
- ◯ 6 Bilateral Support: To be able to walk as far as 25 feet I must have 2 canes or crutches or a walker. I may use a scooter or wheelchair for longer distances.
- ◯ 7 Wheelchair / Scooter: My main form of mobility is a wheelchair. I may be able to stand and/or take one or two steps, but I can't walk 25 feet, even with crutches or a walker.
- ◯ 8 Bedridden: Unable to sit in a wheelchair for more than one hour.

Finally...

Did you encounter any technical difficulties during this survey?

◯ Yes
◯ No
