# Supplementary material for: Impact of the COVID-19 Pandemic on the Personal Networks and Neurological Outcomes of People With Multiple Sclerosis: Cross-Sectional and Longitudinal Case-Control Study
Source: JMIR Public Health Surveill. 2024 Feb 6;10:e45429. doi: 10.2196/45429 (PMC10879979; doi:10.2196/45429)
Supplement: Multimedia Appendix 2 [file publichealth_v10i1e45429_app2.pdf]

SUPPLEMENTAL MATERIAL

Figure S1. Correlation between potential confounders and patient-reported outcomes.

Using a data-driven approach for covariate selection for downstream adjustment, we calculated the Pearson correlation coefficients (R^2, actual value and heatmap intensity shown inside each box) and the P-values (marked as meeting the pre-defined significance threshold) of potential confounding variables in relation to patient-reported outcomes. Covariates were selected for adjustment in downstream analysis if over 70% of participants had the pertinent information (frequency > 70%), the average correlation coefficient across all patient-reported outcomes was over 0.1 ($R^2 > 0.1$), and the P-values met the nominal significance threshold (P<.05).

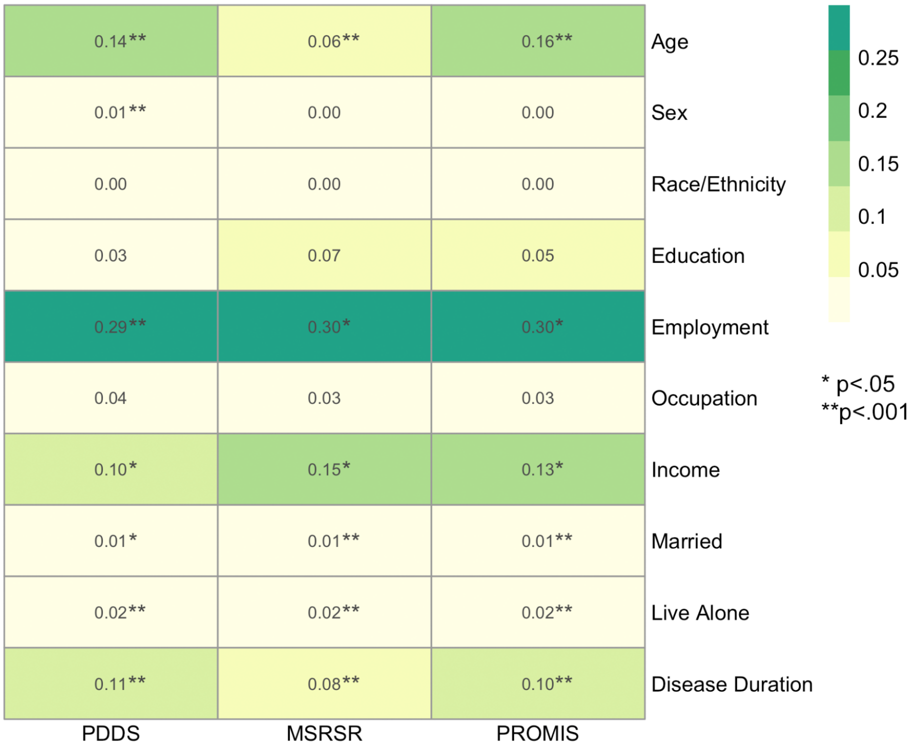

Table S1. Participant characteristics of cross-sectional cohorts by recruitment site.

	Overall: MS	Overall: Control	MS	Control	MS	Control	MS	Control	MS	Control	MS	Control	MS	Control	MS	Control	MS	Control
			Cohort 1[a]	Cohort 1[a]	Cohort 2[b]	Cohort 2[b]	Cohort 3[c]	Cohort 3[c]	Cohort 4[d]	Cohort 4[d]	Cohort 5[e]	Cohort 5[e]	Cohort 6[f]	Cohort 6[f]	Cohort 7[g]	Cohort 7[g]	Cohort 8[h]	Cohort 8[h]
N	1130	1250	282	60	60	165	67	2	129	222	114	34	127	12	290	23	61	732
Age, Mean (SD)	50.7 (12.1)	44.4 (12.1)	50.8 (12.9)	53.1 (13.7)	49.3 (11.5)	53.6 (17.2)	46.7 (11.0)	26.5 (38.9)	49.7 (10.9)	41.6 (9.1)	48.5 (13.5)	52.7 (17.1)	55.4 (11.8)	52.5 (20.1)	51.6 (12.0)	55.5 (15.1)	48.1 (7.4)	41.6 (8.3)
Sex, N (%)																		
Female	925 (81.9)	960 (76.8)	229 (81.2)	31 (51.7)	52 (86.7)	115 (69.7)	51 (76.1)	0 (0.0)	104 (80.6)	183 (82.4)	92 (80.7)	19 (55.9)	101 (79.5)	7 (58.3)	242 (83.4)	16 (69.6)	54 (88.5)	589 (80.5)
Male	203 (18.0)	286 (22.9)	53 (18.8)	28 (46.7)	7 (11.7)	48 (29.1)	16 (23.9)	2 (100.0)	25 (19.4)	39 (17.6)	22 (19.3)	15 (44.1)	25 (19.7)	5 (41.7)	48 (16.6)	7 (30.4)	7 (11.5)	142 (19.4)
Other	2 (0.2)	4 (0.3)	0 (0.0)	1 (1.7)	1 (1.7)	2 (1.2)	0 (0.0)	0 (0.0)	0 (0.0)	0 (0.0)	0 (0.0)	0 (0.0)	1 (0.8)	0 (0.0)	0 (0.0)	0 (0.0)	0 (0.0)	1 (0.1)
Race, N (%)																		
African or African American	46 (4.1)	12 (1.0)	8 (2.8)	0 (0.0)	1 (1.7)	1 (0.6)	7 (10.4)	0 (0.0)	4 (3.1)	2 (0.9)	2 (1.7)	1 (3.0)	6 (4.7)	0 (0.0)	17 (6.0)	0 (0.0)	1 (1.6)	8 (1.1)
American Indian or Alaskan Native or Native Hawaiian	10 (0.9)	6 (0.5)	0 (0.0)	0 (0.0)	0 (0.0)	1 (0.6)	1 (1.5)	0 (0.0)	0 (0.0)	3 (1.4)	7 (6.0)	0 (0.0)	0 (0.0)	0 (0.0)	2 (0.7)	0 (0.0)	0 (0.0)	2 (0.3)
Asian	8 (0.7)	8 (0.6)	0 (0.0)	0 (0.0)	2 (3.3)	2 (1.2)	1 (1.5)	0 (0.0)	1 (0.8)	0 (0.0)	0 (0.0)	2 (6.1)	2 (1.6)	0 (0.0)	1 (0.4)	0 (0.0)	1 (1.6)	4 (0.5)
White	1043 (92.7)	1208 (96.7)	274 (97.2)	60 (100.0)	55 (91.7)	160 (97.0)	50 (74.6)	2 (100.0)	121 (93.8)	213 (95.9)	104 (89.7)	30 (90.9)	119 (93.7)	12 (100.0)	261 (92.2)	23 (100.0)	59 (96.7)	708 (96.7)
Multi-racial	9 (0.8)	11 (0.9)	0 (0.0)	0 (0.0)	1 (1.7)	1 (0.6)	5 (7.5)	0 (0.0)	0 (0.0)	3 (1.4)	3 (2.6)	0 (0.0)	0 (0.0)	0 (0.0)	0 (0.0)	0 (0.0)	0 (0.0)	7 (1.0)
Other	4 (0.4)	3 (0.2)	0 (0.0)	0 (0.0)	1 (1.7)	0 (0.0)	0 (0.0)	0 (0.0)	2 (1.6)	0 (0.0)	0 (0.0)	0 (0.0)	0 (0.0)	0 (0.0)	2 (0.7)	0 (0.0)	0 (0.0)	1 (0.1)
Not Sure	5 (0.4)	1 (0.1)	0 (0.0)	0 (0.0)	0 (0.0)	0 (0.0)	3 (4.5)	0 (0.0)	1 (0.8)	1 (0.5)	0 (0.0)	0 (0.0)	0 (0.0)	0 (0.0)	0 (0.0)	0 (0.0)	0 (0.0)	2 (0.3)
Ethnicity, N (%)																		
Hispanic or Latino	38 (3.4)	31 (2.5)	0 (0.0)	0 (0.0)	5 (8.3)	2 (1.2)	6 (9.0)	0 (0.0)	7 (5.4)	6 (2.7)	8 (6.9)	1 (3.1)	2 (1.6)	0 (0.0)	10 (3.5)	0 (0.0)	0 (0.0)	22 (3.0)
Non- Hispanic	1068 (94.9)	1201 (96.3)	282 (100.0)	60 (100.0)	54 (90.0)	163 (98.8)	55 (82.1)	0 (0.0)	122 (94.6)	213 (95.9)	103 (88.8)	30 (93.8)	122 (96.1)	12 (100.0)	269 (95.1)	23 (100.0)	61 (100.0)	700 (95.8)
Not Sure	19 (1.7)	15 (1.2)	0 (0.0)	0 (0.0)	1 (1.7)	0 (0.0)	6 (9.0)	2 (100.0)	0 (0.0)	3 (1.4)	5 (4.3)	1 (3.1)	3 (2.4)	0 (0.0)	4 (1.4)	0 (0.0)	0 (0.0)	9 (1.2)
Education, N (%)																		

High school graduate	70 (6.2)	35 (2.8)	28 (10.0)	12 (20.0)	4 (6.8)	7 (4.2)	1 (1.6)	0 (0.0)	6 (4.7)	1 (0.5)	7 (6.2)	0 (0.0)	7 (5.5)	0 (0.0)	16 (5.5)	4 (17.4)	1 (1.6)	11 (1.5)
Some college	154 (13.7)	106 (8.5)	47 (16.8)	9 (15.0)	4 (6.8)	16 (9.7)	4 (6.3)	0 (0.0)	18 (14.0)	17 (7.7)	24 (21.2)	3 (8.8)	21 (16.5)	1 (8.3)	31 (10.7)	8 (34.8)	5 (8.2)	52 (7.1)
Associate degree	111 (9.9)	70 (5.6)	43 (15.4)	6 (10.0)	6 (10.2)	10 (6.1)	3 (4.8)	0 (0.0)	14 (10.9)	14 (6.3)	8 (7.1)	4 (11.8)	20 (15.7)	0 (0.0)	13 (4.5)	2 (8.7)	4 (6.6)	34 (4.7)
Bachelor's degree	363 (32.4)	441 (35.3)	81 (28.9)	16 (26.7)	18 (30.5)	61 (37.0)	28 (44.4)	1 (50.0)	36 (27.9)	76 (34.2)	26 (23.0)	8 (23.5)	33 (26.0)	6 (50.0)	114 (39.3)	3 (13.0)	27 (44.3)	270 (36.9)
Graduate degree	418 (37.3)	595 (47.6)	81 (28.9)	17 (28.3)	27 (45.8)	70 (42.4)	27 (42.9)	1 (50.0)	54 (41.9)	114 (51.4)	48 (42.5)	19 (55.9)	44 (34.6)	5 (41.7)	114 (39.3)	6 (26.1)	23 (37.7)	363 (49.7)
Employment, N (%)																		
Employed for wages	544 (48.1)	876 (70.1)	121 (42.9)	30 (50.0)	33 (55.0)	74 (44.8)	36 (53.7)	1 (50.0)	66 (51.2)	169 (76.1)	60 (52.6)	18 (52.9)	47 (37.0)	6 (50.0)	140 (48.3)	4 (17.4)	41 (67.2)	574 (78.4)
Homemaker	63 (5.6)	77 (6.2)	15 (5.3)	0 (0.0)	1 (1.7)	3 (1.8)	4 (6.0)	0 (0.0)	5 (3.9)	16 (7.2)	8 (7.0)	0 (0.0)	7 (5.5)	1 (8.3)	21 (7.2)	0 (0.0)	2 (3.3)	57 (7.8)
Military	2 (0.2)	0 (0.0)	0 (0.0)	0 (0.0)	0 (0.0)	0 (0.0)	0 (0.0)	0 (0.0)	1 (0.8)	0 (0.0)	0 (0.0)	0 (0.0)	0 (0.0)	0 (0.0)	0 (0.0)	0 (0.0)	1 (1.6)	0 (0.0)
Out of work and looking for work	17 (1.5)	27 (2.2)	4 (1.4)	0 (0.0)	1 (1.7)	4 (2.4)	2 (3.0)	0 (0.0)	2 (1.6)	8 (3.6)	2 (1.8)	1 (2.9)	4 (3.1)	0 (0.0)	1 (0.3)	0 (0.0)	1 (1.6)	14 (1.9)
Out of work but not currently looking for work	29 (2.6)	15 (1.2)	7 (2.5)	0 (0.0)	2 (3.3)	4 (2.4)	1 (1.5)	1 (50.0)	5 (3.9)	2 (0.9)	4 (3.5)	0 (0.0)	6 (4.7)	0 (0.0)	4 (1.4)	0 (0.0)	0 (0.0)	8 (1.1)
Retired	187 (16.5)	98 (7.8)	46 (16.3)	16 (26.7)	6 (10.0)	55 (33.3)	8 (11.9)	0 (0.0)	20 (15.5)	2 (0.9)	18 (15.8)	8 (23.5)	30 (23.6)	3 (25.0)	56 (19.3)	7 (30.4)	3 (4.9)	7 (1.0)
Self-employed	63 (5.6)	97 (7.8)	11 (3.9)	5 (8.3)	2 (3.3)	10 (6.1)	3 (4.5)	0 (0.0)	7 (5.4)	16 (7.2)	7 (6.1)	4 (11.8)	5 (3.9)	0 (0.0)	25 (8.6)	1 (4.3)	3 (4.9)	61 (8.3)
Student	12 (1.1)	18 (1.4)	5 (1.8)	0 (0.0)	1 (1.7)	8 (4.8)	2 (3.0)	0 (0.0)	1 (0.8)	4 (1.8)	1 (0.9)	0 (0.0)	0 (0.0)	0 (0.0)	2 (0.7)	0 (0.0)	0 (0.0)	6 (0.8)
Unable to work	190 (16.8)	39 (3.1)	64 (22.7)	9 (15.0)	14 (23.3)	5 (3.0)	9 (13.4)	0 (0.0)	18 (14.0)	5 (2.3)	13 (11.4)	3 (8.8)	27 (21.3)	2 (16.7)	37 (12.8)	11 (47.8)	8 (13.1)	4 (0.5)
Household Income (%)																		
0 to $19,999	74 (7.6)	37 (3.1)	32 (11.7)	8 (13.6)	6 (10.7)	11 (6.9)	*	*	6 (4.9)	5 (2.4)	6 (5.7)	3 (8.8)	7 (7.1)	0 (0.0)	12 (4.7)	3 (20.0)	5 (8.6)	7 (1.0)
$20,000 to $34,999	85 (8.7)	56 (4.7)	26 (9.5)	3 (5.1)	6 (10.7)	17 (10.6)	*	*	10 (8.2)	8 (3.9)	12 (11.3)	1 (2.9)	15 (15.2)	0 (0.0)	13 (5.1)	2 (13.3)	3 (5.2)	25 (3.5)
$35,000 to $49,999	97 (10.0)	75 (6.3)	43 (15.7)	6 (10.2)	8 (14.3)	11 (6.9)	*	*	8 (6.6)	10 (4.8)	8 (7.5)	2 (5.9)	9 (9.1)	0 (0.0)	16 (6.2)	1 (6.7)	5 (8.6)	45 (6.4)
$50,000 to $64,999	92 (9.5)	106 (8.9)	28 (10.2)	6 (10.2)	5 (8.9)	24 (15.0)	*	*	9 (7.4)	20 (9.7)	12 (11.3)	2 (5.9)	15 (15.2)	1 (12.5)	21 (8.2)	0 (0.0)	2 (3.4)	53 (7.5)

$65,000 to $79,999	79 (8.1)	120 (10.1)	26 (9.5)	8 (13.6)	4 (7.1)	16 (10.0)	*	*	14 (11.5)	21 (10.1)	10 (9.4)	3 (8.8)	8 (8.1)	0 (0.0)	12 (4.7)	0 (0.0)	5 (8.6)	72 (10.2)
$80,000 to $94,999	76 (7.8)	94 (7.9)	15 (5.5)	3 (5.1)	2 (3.6)	19 (11.9)	*	*	11 (9.0)	14 (6.8)	6 (5.7)	6 (17.6)	11 (11.1)	1 (12.5)	26 (10.1)	2 (13.3)	5 (8.6)	49 (6.9)
$95,000 to $109,999	80 (8.2)	127 (10.7)	29 (10.6)	6 (10.2)	2 (3.6)	13 (8.1)	*	*	14 (11.5)	22 (10.6)	4 (3.8)	3 (8.8)	11 (11.1)	2 (25.0)	15 (5.8)	0 (0.0)	5 (8.6)	81 (11.5)
$110,000 to $124,999	85 (8.7)	112 (9.4)	26 (9.5)	6 (10.2)	4 (7.1)	15 (9.4)	*	*	7 (5.7)	21 (10.1)	12 (11.3)	3 (8.8)	3 (3.0)	1 (12.5)	26 (10.1)	1 (6.7)	7 (12.1)	65 (9.2)
$125,000 or higher	303 (31.2)	461 (38.8)	49 (17.9)	13 (22.0)	19 (33.9)	34 (21.2)	*	*	43 (35.2)	86 (41.5)	35 (33.0)	10 (29.4)	20 (20.2)	3 (37.5)	116 (45.1)	6 (40.0)	21 (36.2)	309 (43.8)
Married, N (%)	786 (69.9)	841 (67.4)	185 (66.1)	44 (73.3)	37 (62.7)	86 (52.1)	45 (67.2)	1 (50.0)	95 (73.6)	151 (68.0)	84 (73.7)	14 (42.4)	82 (64.6)	11 (91.7)	214 (73.8)	14 (60.9)	44 (74.6)	520 (71.2)
Live Alone, N (%)	156 (14.8)	186 (15.0)	52 (18.5)	11 (19.0)	13 (22.0)	43 (26.1)	*	*	16 (12.6)	27 (12.3)	14 (12.3)	8 (24.2)	20 (15.9)	1 (8.3)	32 (11.0)	6 (26.1)	9 (15.3)	90 (12.3)
Occupation, N (%)																		
Business owner	35 (6.2)	23 (2.4)	9 (6.8)	0 (0.0)	2 (5.7)	3 (3.6)	*	*	5 (6.8)	5 (2.7)	2 (3.0)	1 (4.5)	3 (5.8)	0 (0.0)	11 (6.7)	0 (0.0)	3 (6.8)	14 (2.2)
Executive, manager	100 (17.6)	157 (16.2)	19 (14.4)	5 (14.3)	2 (5.7)	5 (6.0)	*	*	12 (16.4)	31 (16.8)	13 (19.4)	8 (36.4)	5 (9.6)	1 (16.7)	38 (23.0)	2 (40.0)	11 (25.0)	105 (16.5)
Laborer, unskilled worker	7 (1.2)	4 (0.4)	4 (3.0)	1 (2.9)	0 (0.0)	0 (0.0)	*	*	0 (0.0)	0 (0.0)	0 (0.0)	0 (0.0)	2 (3.8)	0 (0.0)	0 (0.0)	0 (0.0)	1 (2.3)	3 (0.5)
Machine operator, inspector, bus/cab driver	1 (0.2)	0 (0.0)	0 (0.0)	0 (0.0)	0 (0.0)	0 (0.0)	*	*	0 (0.0)	0 (0.0)	1 (1.5)	0 (0.0)	0 (0.0)	0 (0.0)	0 (0.0)	0 (0.0)	0 (0.0)	0 (0.0)
Mechanic, electrician, skilled worker	6 (1.1)	14 (1.4)	0 (0.0)	0 (0.0)	0 (0.0)	1 (1.2)	*	*	0 (0.0)	3 (1.6)	1 (1.5)	0 (0.0)	1 (1.9)	0 (0.0)	4 (2.4)	0 (0.0)	0 (0.0)	10 (1.6)
Other	80 (14.1)	129 (13.3)	19 (14.4)	3 (8.6)	5 (14.3)	17 (20.2)	*	*	11 (15.1)	27 (14.6)	12 (17.9)	4 (18.2)	6 (11.5)	0 (0.0)	21 (12.7)	0 (0.0)	6 (13.6)	78 (12.3)
Professional	282 (49.6)	545 (56.1)	66 (50.0)	19 (54.3)	22 (62.9)	46 (54.8)	*	*	38 (52.1)	106 (57.3)	33 (49.3)	8 (36.4)	28 (53.8)	4 (66.7)	73 (44.2)	2 (40.0)	22 (50.0)	360 (56.7)
Sales or clerical worker	54 (9.5)	84 (8.6)	14 (10.6)	5 (14.3)	4 (11.4)	10 (11.9)	*	*	5 (6.8)	10 (5.4)	5 (7.5)	1 (4.5)	7 (13.5)	1 (16.7)	18 (10.9)	1 (20.0)	1 (2.3)	56 (8.8)
Service worker	3 (0.5)	16 (1.6)	1 (0.8)	2 (5.7)	0 (0.0)	2 (2.4)	*	*	2 (2.7)	3 (1.6)	0 (0.0)	0 (0.0)	0 (0.0)	0 (0.0)	0 (0.0)	0 (0.0)	0 (0.0)	9 (1.4)
Network Size, Mean (SD)	6.8 (4.2)	6.8 (3.9)	6.1 (3.6)	5.8 (3.7)	6.9 (4.I4)	7.0 (4.5)	8.2 (4.6)	4.5 (0.7)	7.4 (4.3)	7.2 (4.0)	7.1 (3.9)	5.6 (3.5)	5.4 (3.4)	7.2 (3.2)	7.4 (4.8)	7.3 (5.1)	6.6 (4.3)	6.7 (3.7)

PDDS, Mean (SD)	1.9 (2.1)	N/A	2.2 (2.2)	N/A	2.1 (1.9)	N/A	1.6 (2.0)	N/A	1.8 (2.3)	N/A	1.4 (1.9)	N/A	2.2 (2.4)	N/A	1.6 (2.0)	N/A	1.3 (1.9)	N/A
MSRS-R, Mean (SD)	7.6 (5.5)	N/A	8.0 (5.5)	N/A	8.8 (5.5)	N/A	*	N/A	7.4 (5.9)	N/A	6.7 (5.6)	N/A	8.7 (5.6)	N/A	7.0 (5.0)	N/A	6.5 (5.8)	N/A
PROMIS Physical Function T-score, Mean (SD)	46.4 (10.8)	56.3 (9.0)	45.3 (10.6)	51.2 (10.4)	45.8 (11.5)	53.5 (8.9)	48.9 (10.3)	42.7 (4.5)	46.1 (11.9)	59.2 (8.0)	47.3 (10.3)	45.5 (10.9)	43.7 (10.6)	45.5 (7.8)	47.7 (10.8)	42.3 (11.2)	48.3 (9.2)	57.5 (7.9)

a. Cross-sectional cohorts include Cohort 1 and 2 (University of Pittsburgh: clinic-based cohort, MSReCOV recruitment), Cohort 3 and 4 (Columbia University: clinic-based cohort, MSReCOV recruitment), Cohort 5 (Yale University), Cohort 6 (University of Buffalo Medical Center), Cohort 7 (University of Pennsylvania), and Cohort 8 (GEMS cohort).
b. PDDS: Patient-Determined Disease Steps.
c. MSRS-R: Multiple Sclerosis Rating Scale-Revised.
d. PROMIS: Patient-Reported Outcomes Measurement Information System Physical Function.
e. Abbreviation: * - Data unavailable, N/A - not applicable.

Table S2. Cross-sectional analysis of personal social network features in relation to PROMIS Physical Function during the COVID-19 pandemic in controls.

	N	Beta [a]	95% CI (lower)	95% CI (upper)	P-value [b]
Network Structure					
Size	1047	0.172	0.048	0.295	.007
Density	1005	-0.693	-2.689	1.304	.496
Constraint	1005	-0.034	-0.063	-0.005	.020
Effective Size	1005	0.313	0.018	0.608	.038
Maximum Degree	1005	0.383	0.141	0.626	**.002**
Mean Degree	1005	0.326	0.025	0.628	.034
Network Composition					
Percent Kin	1022	-1.065	-2.800	0.669	.228
Standard Deviation of Age	760	-0.025	-0.123	0.073	.619
Diversity of Sex	1022	0.596	-1.021	2.212	.470
Diversity of Race	1020	2.844	-0.013	5.702	.051
Percent contacted weekly or less	1022	-1.522	-3.840	0.796	.198
Percent known for less than 6 years	1022	0.713	-1.581	3.007	.542
Percent who live over 15 miles away	1022	-0.741	-2.541	1.059	.419
Percent who drink	1022	-1.138	-2.917	0.642	.210
Percent who smoke	1022	-4.846	-7.919	-1.774	**.002**
Percent non exercisers	1022	-1.574	-3.212	0.064	.060
Percent bad diet	1022	-2.556	-4.267	-0.845	.003
Percent who have a negative health influence	1022	-5.707	-7.405	-4.010	**<.001**

a. Adjusted for potential confounders including age, employment, and income. Disease duration was not adjusted since it does not apply to controls.
b. Bolded P-values meet the significance threshold of P<.002 (α = 0.05, corrected for 18 comparisons).

Table S3. Longitudinal examination of the association between changes in personal social network features (pandemic values minus pre-pandemic baseline) in relation to patient-reported outcomes in people with multiple sclerosis during the pandemic.

	PDDS [a]					MSRS-R [b]					PROMIS [c]				
	N	Beta [d]	95% CI (lower)	95% CI (upper)	P-value [d]	N	Beta [d]	95% CI (lower)	95% CI (upper)	P-value [d]	N	Beta [d]	95% CI (lower)	95% CI (upper)	P-value [d]
Network Structure															
Size	185	-0.004	-0.063	0.055	.892	185	-0.071	-0.211	0.070	.324	178	0.040	-0.253	0.334	.786
Density	166	-0.571	-1.951	0.809	.415	166	-1.715	-5.074	1.645	.315	162	5.581	-0.851	12.014	.088
Constraint	166	-0.009	-0.026	0.008	.300	166	-0.012	-0.053	0.029	.574	162	0.084	0.004	0.165	.041
Effective Size	166	0.027	-0.172	0.225	.791	166	0.020	-0.464	0.504	.935	162	-0.610	-1.554	0.333	.203
Maximum Degree	166	0.011	-0.122	0.143	.874	166	0.005	-0.317	0.327	.975	162	-0.248	-0.88	0.385	.440
Mean Degree	166	0.012	-0.156	0.179	.891	166	-0.055	-0.464	0.354	.791	162	-0.234	-1.048	0.58	.571
Network Composition															
Percent Kin	171	-0.952	-2.225	0.320	.141	171	-0.849	-3.956	2.257	.590	166	7.697	1.737	13.657	.012
Standard Deviation of Age	139	-0.069	-0.159	0.022	.129	139	-0.137	-0.399	0.125	.290	134	0.016	-0.496	0.528	.945
Diversity of Sex	170	0.181	-0.728	1.089	.695	170	-1.540	-3.700	0.621	.161	165	-1.049	-5.361	3.262	.631
Diversity of Race	170	-1.456	-3.754	0.842	.213	170	-3.522	-9.164	2.119	.219	165	3.909	-7.267	15.085	.490
Percent contacted weekly or less	171	0.183	-1.103	1.469	.779	171	-0.442	-3.562	2.679	.780	166	-2.732	-8.807	3.343	.375
Percent known for less than 6 years	171	1.080	-0.569	2.729	.198	171	1.071	-2.949	5.091	.599	166	-5.856	-13.668	1.956	.141
Percent who live over 15 miles away	171	-0.396	-1.319	0.527	.398	171	-0.901	-3.141	1.339	.428	166	2.366	-2.188	6.921	.306
Percent who drink	143	-0.56	-1.938	0.818	.423	143	-1.89	-4.973	1.192	.227	143	2.833	-3.713	9.379	.393
Percent who smoke	171	0.475	-0.904	1.854	.497	171	-3.319	-6.626	-0.012	.049	166	0.514	-6.179	7.207	.880

Percent non exercisers	171	0.439	-0.394	1.271	.299	171	1.025	-0.995	3.045	.318	166	2.13	-1.831	6.09	.290
Percent bad diet	143	0.202	-1.072	1.476	.754	143	-0.434	-3.294	2.427	.764	143	2.418	-3.626	8.461	.430
Percent who have a negative health influence	143	0.113	-0.852	1.077	.817	143	0.253	-1.912	2.418	.817	143	-1.144	-5.725	3.437	.622

a. PDDS: Patient-Determined Disease Steps.
b. MSRS-R: Multiple Sclerosis Rating Scale-Revised.
c. PROMIS: Patient-Reported Outcomes Measurement Information System, Physical Function.
d. Adjusted for potential confounders, including age, disease duration, employment, income, study cohort, and time lapse between pre-pandemic and pandemic assessment. None of the P-values meet the significance threshold of $P<.00092$ ($\alpha = 0.05$, corrected for 54 comparisons).

Table S4. Moderation analysis assessing the influence of multiple sclerosis (MS) diagnosis on the direction and strength of the association between personal social network features and PROMIS Physical Function during the COVID-19 pandemic.

		**Association Between Network Feature and PROMIS Physical Function**				**Moderating Effect of MS Diagnosis on Association Between Network Feature and PROMIS Physical Function**			
	N	Beta	95% CI (lower)	95% CI (upper)	P-value [a]	Beta	95% CI (lower)	95% CI (upper)	P-value [a]
Network Structure									
Size	1985	0.194	-0.112	0.5	.214	0.035	-0.157	0.226	.723
Density	1896	-1.047	-6.032	3.938	.680	0.389	-2.77	3.547	.809
Constraint	1896	-0.067	-0.138	0.004	.064	0.022	-0.022	0.067	.325
Effective Size	1896	0.423	-0.325	1.17	.267	-0.018	-0.502	0.466	.943
Maximum Degree	1896	0.784	0.179	1.389	.011	-0.27	-0.654	0.115	.169
Mean Degree	1896	0.709	-0.036	1.454	.062	-0.282	-0.745	0.182	.233
Network Composition									
Percent Kin	1927	-0.234	-4.554	4.085	.915	-0.924	-3.656	1.808	.507
Standard Deviation of Age	1321	-0.019	-0.258	0.22	.876	0.002	-0.147	0.151	.976
Diversity of Sex	1921	0.915	-3.117	4.947	.656	-0.351	-2.925	2.22	.789
Diversity of Race	1906	8.788	1.409	16.167	.019	-5.878	-10.889	-0.868	**.022**
Percent contacted weekly or less	1927	-0.547	-6.354	5.259	.853	0.329	-3.407	4.064	.863
Percent known for less than 6 years	1927	-3.821	-9.437	1.796	.182	2.744	-0.989	6.477	.150
Percent who live over 15 miles away	1927	-5.434	-9.91	-0.957	.017	4.662	1.811	7.512	**.001**
Percent who drink	1927	-5.679	-10.262	-1.096	.015	4.697	1.653	7.741	**.002**
Percent who smoke	1927	-7.724	-15.198	-0.25	.043	1.136	-3.451	5.723	.627
Percent non exercisers	1927	-3.508	-7.576	0.56	.091	1.116	-1.449	3.682	.394

Percent bad diet	1927	-1.633	-5.924	2.658	.456	-0.972	-3.766	1.821	.495
Percent who have a negative health influence	1927	-11.028	-15.237	-6.819	<.001	4.427	1.786	7.069	**.001**

a. Bolded P-values met the significance threshold of P<.05.

Table S5. Moderation analysis assessing the influence of having a multiple sclerosis (MS) diagnosis on the direction and strength of the association between personal social network features and PROMIS Physical Function during the COVID-19 pandemic in people with multiple sclerosis and control participant subgroups.

	MS		Control	
	Slope [95% CI]	P-value	Slope [95% CI]	P-value
Diversity of Race	-2.968 [-6.893, 0.956]	.138	2.910 [-0.224, 6.044]	.068
Percent who live over 15 miles away	3.890 [1.852, 5.928]	**<.001**	-0.772 [-2.765, 1.221]	.448
Percent who drink	3.716 [1.392, 6.039]	**.002**	-0.982 [-2.966, 1.003]	.332
Percent who have a negative health influence	-2.174 [-4.019, -0.329]	**.021**	-6.601 [-8.494, -4.708]	**<.001**

a. Bolded P-values met the significance threshold of $P<.05$.
